# Time-Resolved Imaging of High Mass Proteins and Metastable Fragments Using Matrix-Assisted Laser Desorption/Ionization, Axial Time-of-Flight Mass Spectrometry, and TPX3CAM

**DOI:** 10.1021/acs.analchem.2c04480

**Published:** 2022-12-27

**Authors:** Anjusha Mathew, Joel D. Keelor, Gert B. Eijkel, Ian G. M. Anthony, Jingming Long, Jord Prangsma, Ron M. A. Heeren, Shane R. Ellis

**Affiliations:** † Maastricht MultiModal Molecular Imaging (M4i) Institute, Division of Imaging Mass Spectrometry (IMS), Maastricht University, 6229 ER Maastricht, The Netherlands; ‡ Amsterdam Scientific Instruments (ASI), Science Park 106, 1098 XG Amsterdam, The Netherlands; § Molecular Horizons and School of Chemistry and Molecular Bioscience, University of Wollongong, NSW 2522, Wollongong, Australia

## Abstract

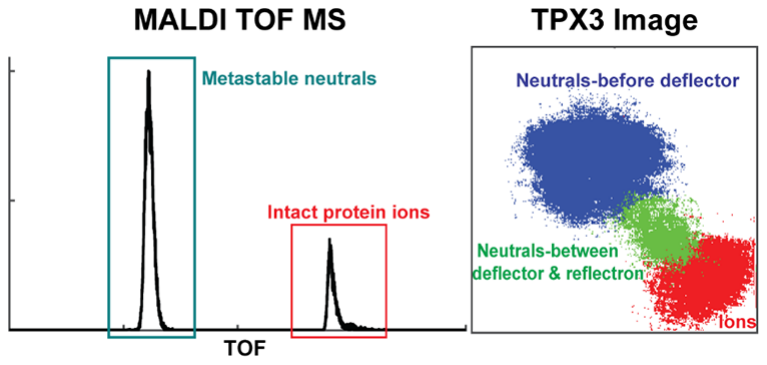

The Timepix (TPX) is a position- and time-sensitive pixelated charge detector that can be coupled with time-of-flight mass spectrometry (TOF MS) in combination with microchannel plates (MCPs) for the spatially and temporally resolved detection of biomolecules. Earlier generation TPX detectors used in previous studies were limited by a moderate time resolution (at best 10 ns) and single-stop detection for each pixel that hampered the detection of ions with high mass-to-charge (*m*/*z*) values at high pixel occupancies. In this study, we have coupled an MCP-phosphor screen-TPX3CAM detection assembly that contains a silicon-coated TPX3 chip to a matrix-assisted laser desorption/ionization (MALDI)-axial TOF MS. A time resolution of 1.5625 ns, per-pixel multihit functionality, simultaneous measurement of TOF and time-over-threshold (TOT) values, and kHz readout rates of the TPX3 extended the *m*/*z* detection range of the TPX detector family. The detection of singly charged intact Immunoglobulin M ions of *m*/*z* value approaching 1 × 10^6^ Da has been demonstrated. We also discuss the utilization of additional information on impact coordinates and TOT provided by the TPX3 compared to conventional MS detectors for the enhancement of the quality of the mass spectrum in terms of signal-to-noise (S/N) ratio. We show how the reduced dead time and event-based readout in TPX3 compared to the TPX improves the sensitivity of high *m*/*z* detection in both low and high mass measurements (*m*/*z* range: 757–970,000 Da). We further exploit the imaging capabilities of the TPX3 detector for the spatial and temporal separation of neutral fragments generated by metastable decay at different locations along the field-free flight region by simultaneous application of deflection and retarding fields.

## Introduction

The Timepix (TPX) detector family consists of position- and time-sensitive pixelated charge detectors, with each pixel capable of recording both the arrival time and impact position coordinates of impinging particles.^1−3^ Although TPX detectors originate from high-energy physics, the integration of TPX with microchannel plate (MCP) amplifiers has enabled the detection of low-energy particles^4−6^ that extended their scope to biomolecular mass spectrometry (MS).^7−21^ MCP-TPX detection systems were previously coupled to time-of-flight (TOF) mass spectrometers equipped with nano/micro-electrospray ionization (ESI), matrix-assisted laser desorption/ionization (MALDI), or secondary ion MS (SIMS) sources, where the arrival time information at the TPX detector was used for the generation of the mass spectrum. The additional impact position information provided by the TPX compared to conventional MS detectors has previously been utilized to obtain insight into the ion transport properties and ion optical processes within the MS,^14,16,18,20^ as well as to improve the spatial resolution and throughput of MS imaging via stigmatic ion imaging.^7,9,10,13,15,19^ The TPX detector used for previous studies was limited by a moderate time resolution (at best 10 ns) and single-stop detection for each pixel that can result in a low intensity of higher mass-to-charge (*m*/*z*) signals (longer flight time) due to the occupancy of pixels by earlier arriving ions.^1^

In this study, we have coupled an MCP-phosphor screen-TPX3CAM detection assembly^22−24^ that contains a silicon (Si)-coated TPX3 chip, the successor of the TPX, for the first time to a MALDI-axial TOF MS (Bruker Ultraflex III). The TPX3 offers a time resolution of 1.5625 ns, allows simultaneous measurement of time-of-arrival (TOA) and time-over-threshold (TOT) values, and operates under data-driven readout mode.^2^ Unlike the previous MCP-bare TPX single/quad systems that had to be placed in vacuum, the additional signal conversion steps in MCP-phosphor screen-TPX3CAM detection assembly allowed the TPX3CAM to be installed at atmospheric pressure that brings considerable flexibility, elimination of several elements, and flexible mapping between phosphor screen and sensor area.

Previous investigations conducted on the Ultraflex III MS coupled with MCP-TPX assembly and the modified LCT (nanoESI-orthogonal acceleration reflectron TOF) MS coupled with HV-floating TPX quad system demonstrated the ability of TPX to detect intact protein ions with *m*/*z* values up to 400,000 Da^11^ and multiply charged non-covalent protein complexes of molecular weight up to 800,000 Da (*m*/*z* < 13,000 Da).^20,21^ Here, we extend the *m*/*z* detection range of TPX family up to 970,000 Da by the measurement of single-charged intact immunoglobulin M (IgM) ions using the MCP-phosphor screen-TPX3CAM assembly, which has previously been used with great success in velocity map imaging to investigate small-molecule reaction dynamics.^25−32^ In addition, we demonstrate the utilization of multiple information provided by TPX3 such as TOA, pixel coordinates, and TOT for better visualization of the mass spectrum. We discuss how the reduced dead time and event-based readout in TPX3 compared to the TPX enhances both the low and high mass measurements (*m*/*z* mass range: 757–970,000 Da) and allows the operation of the whole system 10 times faster. These capabilities of the TPX3 detector allowed us to explore the spatial detection and separation of precursor ions and neutral fragments formed via metastable decay at the different locations within the field-free TOF tube. These spatio-temporal studies provide insights into the molecular processes, such as post-source decay (PSD), that occur in axial TOF systems.

## Materials and Methods

### Materials

Insulin chain B oxidized (3.5 kDa) from bovine pancreas, insulin (5.7 kDa) from bovine pancreas, cytochrome *c* (12.4 kDa) from equine heart, myoglobin (17.6 kDa) from equine heart, immunoglobulin G (IgG, ∼150 kDa) from human serum, immunoglobulin A (IgA, ∼400 kDa) from human colostrum, α-cyano-4-hydroxycinnamic acid (CHCA), sinapinic acid (SA), and trifluoroacetic acid (TFA) were all purchased from Sigma-Aldrich (Zwijndrecht, the Netherlands). The peptide calibration standard II (*m*/*z* range: 700–3200 Da), protein calibration standard I (*m*/*z* range: 5–18 kDa), and protein calibration standard II (*m*/*z* range: 10–70 kDa) were purchased from Bruker GmbH (Bremen, Germany), and C450 IgM MALDI MS calibration kit (∼970 kDa) was purchased from CovalX (Zürich, Switzerland). Acetonitrile and liquid chromatography mass spectrometry (LC-MS) grade water were purchased from Biosolve (Valkenswaard, the Netherlands).

### Sample Preparation

Insulin chain B, insulin, cytochrome *c*, myoglobin, IgG, and IgA were prepared as 0.5–2 mg/mL solution in water. One tube of peptide calibration standard II/protein calibration standard I/protein calibration standard II was dissolved in 125 μL 0.1% TFA solvent according to the manufacturer’s instructions. The SA was prepared as a 20 mg/mL solution in 50% acetonitrile + 0.1% TFA. The CHCA matrix was prepared as 10 mg/mL solution in 70% acetonitrile + 0.2% TFA. The prepared aliquots of proteins/peptides were mixed 1:1 (v/v) with matrix solutions. Then, 1 μL aliquots of each analyte/matrix solution were deposited onto a stainless steel target plate and air-dried prior to the MS analysis.

### TPX3CAM

The TPX3CAM (Amsterdam Scientific Instruments, Amsterdam, the Netherlands) is a fast optical imager based on a specialized Si sensor bump-bonded to the Timepix3 application-specific integrated circuit (TPX3 ASIC) readout chip (Medipix3 collaboration, CERN, Geneva, Switzerland).^2,24,25^ The 300 μm thick silicon sensor has a thin entrance window and anti-reflective coating, and provides an enhanced quantum efficiency (QE) of about 90% over the wavelength range 400–1000 nm. The TPX3 chip is produced in 130 nm CMOS technology and consists of a 256 × 256 pixel matrix having a pixel pitch of 55 μm and dimensions of 1.4 × 1.4 cm^2^. Unlike its precursor TPX chip,^1^ where the readout is frame-based, the readout from TPX3 is data-driven, whereby data is immediately sent out upon the activation of each pixel. Each pixel has a customizable energy threshold level that determines when a hit is registered. If a signal causes a crossing of this threshold, then the hit is registered along with the pixel coordinates, time-of-hit/arrival (TOA), and time taken for the signal to fall below the threshold, which is referred to as the time-over-threshold (TOT). The light detection threshold is about 600–800 photons per pixel, depending on the wavelength. The dead time of individual pixels to process and store the information after they were hit is about 475 ns plus the corresponding TOT. TOA is recorded in a 14-bit register operating at 40 MHz giving a temporal resolution of 25 ns, and improved further to 1.5625 ns using the local 4-bit counter operating at 640 MHz. The data from the TPX3 ASIC is acquired by Speedy PIxel Detector Readout (SPIDR) system (Nikhef, Amsterdam, the Netherlands), which provides both 10 and 1 Gbps ethernet interfaces, and the former can deal with high data output of 80 Mhits per chip per second.^33^ The SPIDR has an internal time-to-digital converter (TDC) which is able to time-stamp incoming digital pulses with 260 ps precision synchronously with the TPX3 hits. This feature is useful to provide an external time reference.

### Mass Spectrometer and Detection System

**Figure 1:**
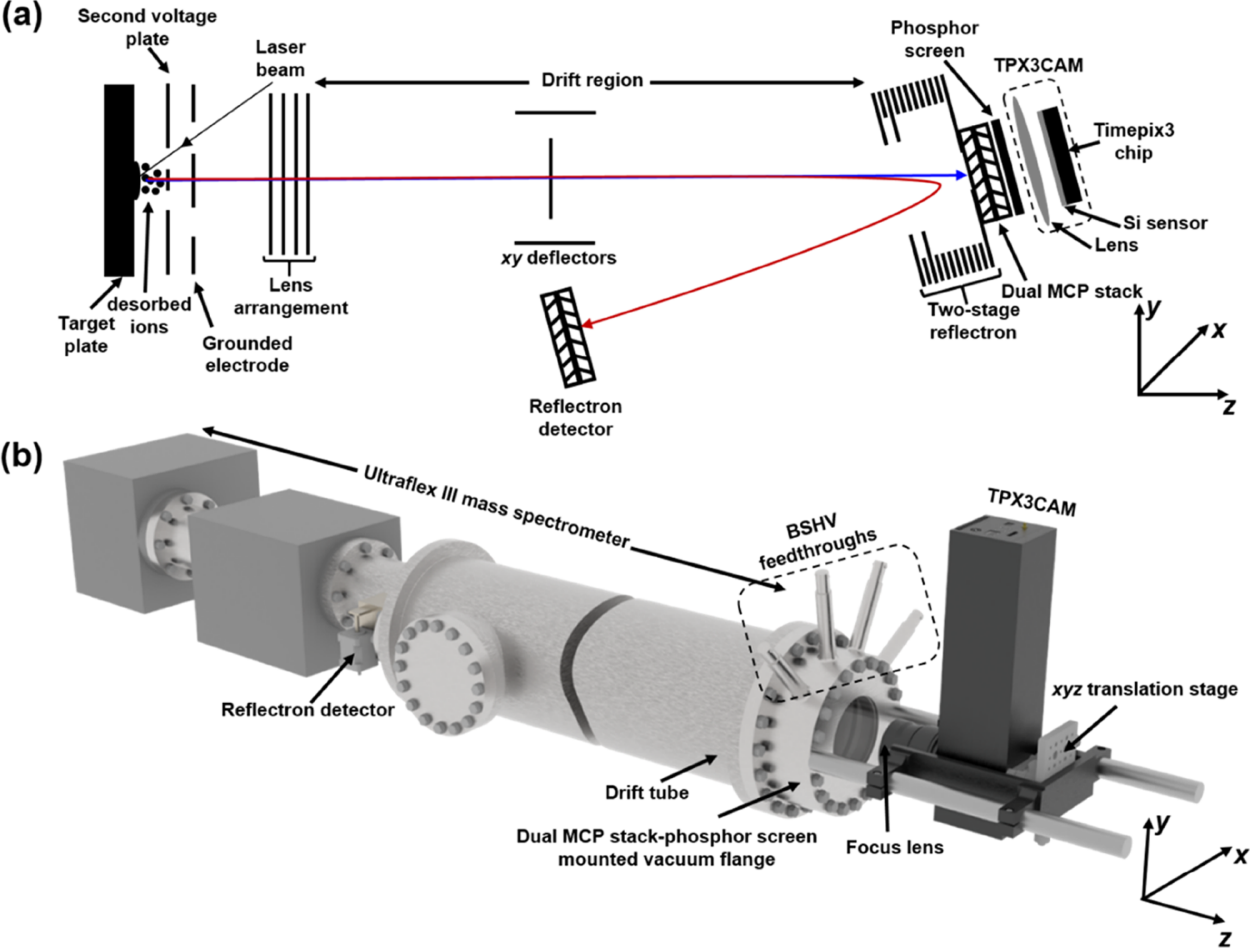
(a) Schematic of the ion optics of Ultraflex III MS. Conventional linear detector is replaced by the MCP-phosphor screen-TPX3CAM assembly. Ions are accelerated in the *z*-direction and sent either to the linear imaging detector (blue trace) or to the standard reflectron detector (red trace). (b) Mechanical schematic of Ultraflex MS coupled with the MCP-phosphor screen-TPX3CAM assembly. MCP-phosphor screen assembly is mounted to the vacuum flange that is positioned at the end of the drift tube, and the TPX3CAM is kept at atmospheric pressure via support rods behind the flange.

All experiments were performed on an Ultraflex III MALDI TOF MS (Bruker Daltonik GmbH, Bremen, Germany)^14^ equipped with a TPX3CAM detection assembly (Figure 1). The MALDI ions generated by the Smartbeam 355 nm Nd:YAG laser are extracted, accelerated, and time-focused through a two-stage acceleration region (target plate, second voltage plate, and a ground electrode) employing delayed ion extraction before passing through a lens system and entering the drift region (Figure 1a). Ions can either be detected in the linear/axial mode or in reflectron mode by the reflection of ions using a two-stage reflectron. The reflectron voltage can also be used to apply a retarding field to ions prior to axial mode detection. The drift region is equipped with a deflection unit that allows deflection of ions in a plane perpendicular to the flight direction.

The conventional Ultraflex linear detector has been replaced by a MCP-phosphor screen-TPX3CAM detection assembly. The mechanical schematic of the detection system coupled with the Ultraflex III MS is shown in Figure 1b. The dual chevron MCP stack generates secondary electrons based on the impinging particle properties. The P47 phosphor screen placed behind the MCPs produces fast flashes of light when impinged by the MCP electrons, which are imaged and time-stamped by the sensor in the TPX3CAM. The two imaging MCPs (40 mm quality diameter, 8° bias angle, 12 μm channel center-to-center spacing, 10 μm pore size, 4 × 10^6^ electron gain at 2000 V) and phosphor screen (40 mm quality diameter, maximum emission at 430 nm, rise time 7 ns, decay time 70 ns)^34^ were mounted to a stainless steel vacuum flange (Photonis USA, Sturbridge) that is equipped with four BSHV feedthroughs. The phosphor screen feedthrough is rated up to 7.5 kV, and the other three are rated up to 5 kV (two MCPs and one spare). The signals from the MCP differential current and phosphor screen current were capacitively decoupled and recorded simultaneously with the TPX3 signal using a fast oscilloscope (∼500 MHz and 4 GS/s, LeCroy LT372). The TPX3CAM with 50 mm f/0.95 lens and 25 mm c-mount extension was installed outside of the vacuum via two support rods ∼55 mm from the vacuum flange. When compared to the previously employed MCP-TPX arrangement, where the entire detecting assembly was placed in vacuum, the TPX3CAM, which is completely decoupled from the rest of the setup, brings considerable flexibility. Furthermore, the direct electron detection approach in the previous studies required close proximity of the MCP and TPX chip (∼2 mm) and/or floating detection assembly and readout electronics at high voltages (kV range) that resulted in severe complications of the design as well as the addition of several other elements.^12,14,20^ Lastly, the optical approach employed here allows flexible mapping between the phosphor screen and TPX3 sensor by magnification/demagnification using appropriate lenses. In this study, the TPX3CAM has been operated in TOF mode, in which the arrival time of each particle (along with TOT and pixel coordinates) is measured with respect to an external trigger. The TPX3 and internal TDC of the SPIDR were triggered at a rate of 10–100 Hz using the laser pulse photodiode signal through the four channels of the digital pulse and delay generator (DG535, Stanford Research Systems, USA). The falling-edge pulse from channels A and B that defines the TOF window was sent to TPX3 through a trigger box, whereas the pulse signal from channels C and D was fed directly to the TDC. The data acquisition parameters are listed in Table S1. All parameters except the linear detection assembly voltages, pulse generator, and oscilloscope settings were defined via FlexControl 3.4 software (Bruker Daltonik GmbH, Bremen, Germany). External power supplies from AMOLF, Amsterdam, the Netherlands, and FuG Elektronik GmbH, Schechen, Germany were used to define the MCPs and phosphor voltages, respectively. TOF to *m*/*z* calibration was performed using the second-order polynomial function generated from the TOF-*m*/*z* conversion curve using 10 samples that encompasses an *m*/*z* range from 750 to 970,000 (Figure S1). All of the TOF data were acquired using an initial acceleration voltage (target plate voltage) of 25 kV.

### Data Acquisition and Analysis

The SoPhy (Software for Physics) software package version 1.6.3 was used for the TPX3CAM control and data acquisition (Amsterdam Scientific Instruments, Amsterdam, the Netherlands). The raw files were subsequently analyzed using open-source Python 3.7.6 with Spyder IDE environment from Anaconda (Anaconda 3, 236, Anaconda Inc., Texas, USA) and GUI built in MATLAB (R2019b, MathWorks Inc., Natick, USA).

**Figure 2:**
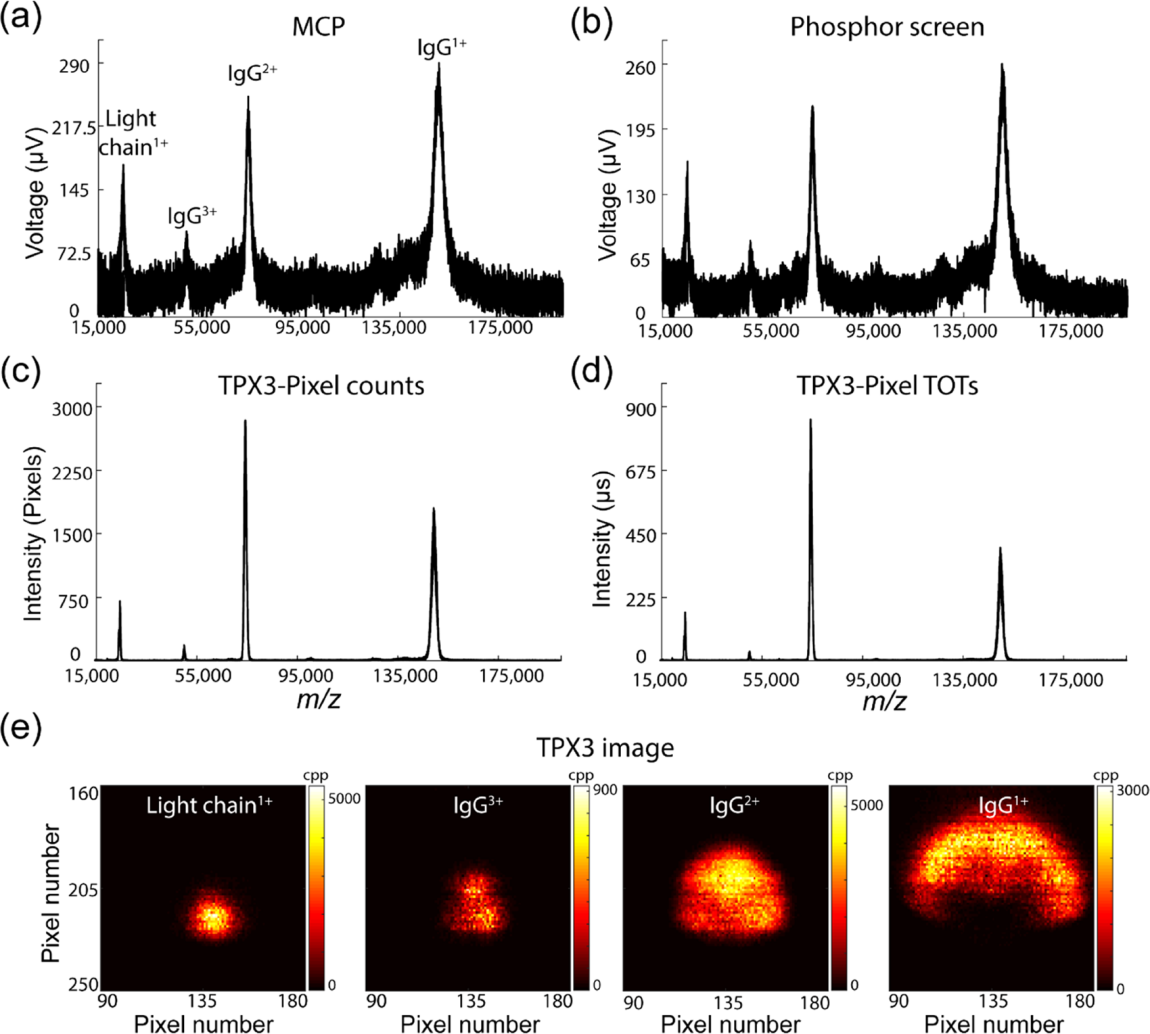
MALDI mass spectra of IgG (in SA matrix) acquired simultaneously from MCP (a), phosphor screen (b), and TPX3 (c, d) detectors for 5000 laser shots. The signal intensity in each case represents different parameters. In panels (a) and (b), the mass spectrum is plotted by integrating the voltage pulses corresponding to each ion event using an oscilloscope, with a sampling rate of 100 MHz. TPX3 data are plotted by summing up the number of pixels activated for each ion event (c) and TOT values corresponding to each pixel hit (d) across 1.5625 ns. (e) *m*/*z*-resolved TPX3 images of LC^1+^, IgG^3+^, IgG^2+^, and IgG^1+^ ions (cpp = counts per pixel). Data acquisition parameters are listed in Table S1 (Supporting Information).

**Figure 3:**
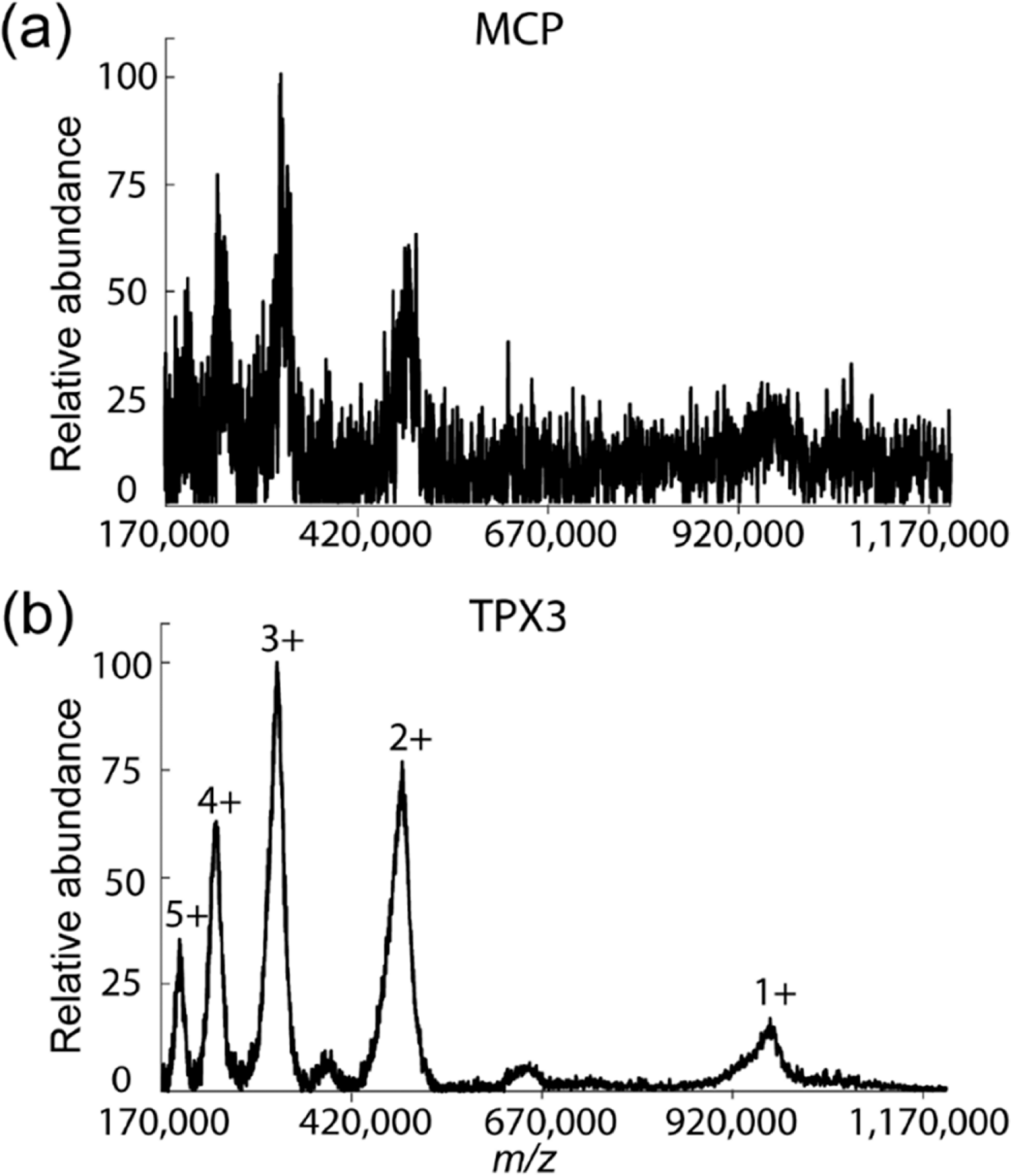
MALDI MS spectra of IgM (in SA matrix) acquired simultaneously on MCP (a) and TPX3 (b) channels for 5000 laser shots. The TPX3 spectrum is generated by summing up the number of pixels activated for each TOA bin. The MCP spectrum was recorded at a 100 MHz digitization rate using an oscilloscope, whereas TPX3 data was acquired using time bins of 1.5625 ns. Both spectra were resampled to 100 ns and baseline subtracted. Data acquisition parameters are listed in Table S1 (Supporting Information).

**Figure 4:**
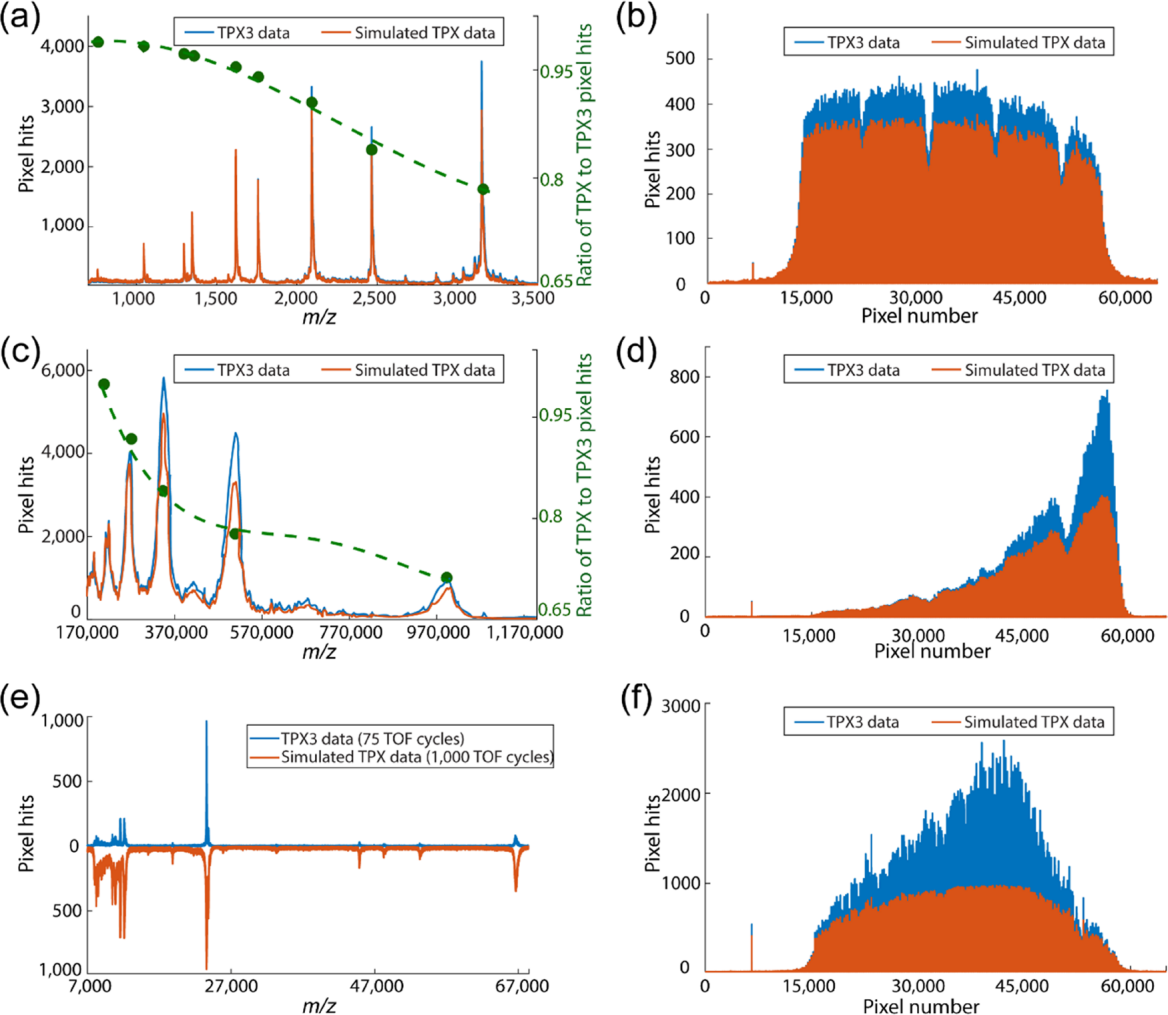
Mass spectra (a, c) and pixel histograms (b, d) plotted in event-based (TPX3, blue trace) and frame-based (TPX, orange trace) modes for two different mass ranges: Bruker peptide calibration standard II (*m*/*z* range: 700–3200 Da, data 1 (top), in CHCA matrix) and IgM (*m*/*z* range: 190–970 kDa, data 2 (middle), in SA matrix) for 1000 laser shots. Green trace in (a) and (c) plots the ratio of signal intensity in TPX and TPX3 modes. Bruker protein calibration standard II (*m*/*z* range: 10–70 Da, data 3 (bottom), in SA matrix) mass spectrum (e) plotted in event-based mode by the accumulation of 75 TOF cycles and in frame-based mode by the accumulation of 1000 laser shots, and pixel histograms (f) plotted for 1000 laser shots in both modes. The spectra are generated by summing up the number of pixels activated for each 1.5625 TOA bin. The pixel histograms are generated by summing up the frequency of pixel activation of each pixel in the 256 × 256 TPX3 pixel array (65,536 pixels) for 1000 laser shots. Data acquisition parameters are listed in Table S1 (Supporting Information).

**Figure 5:**
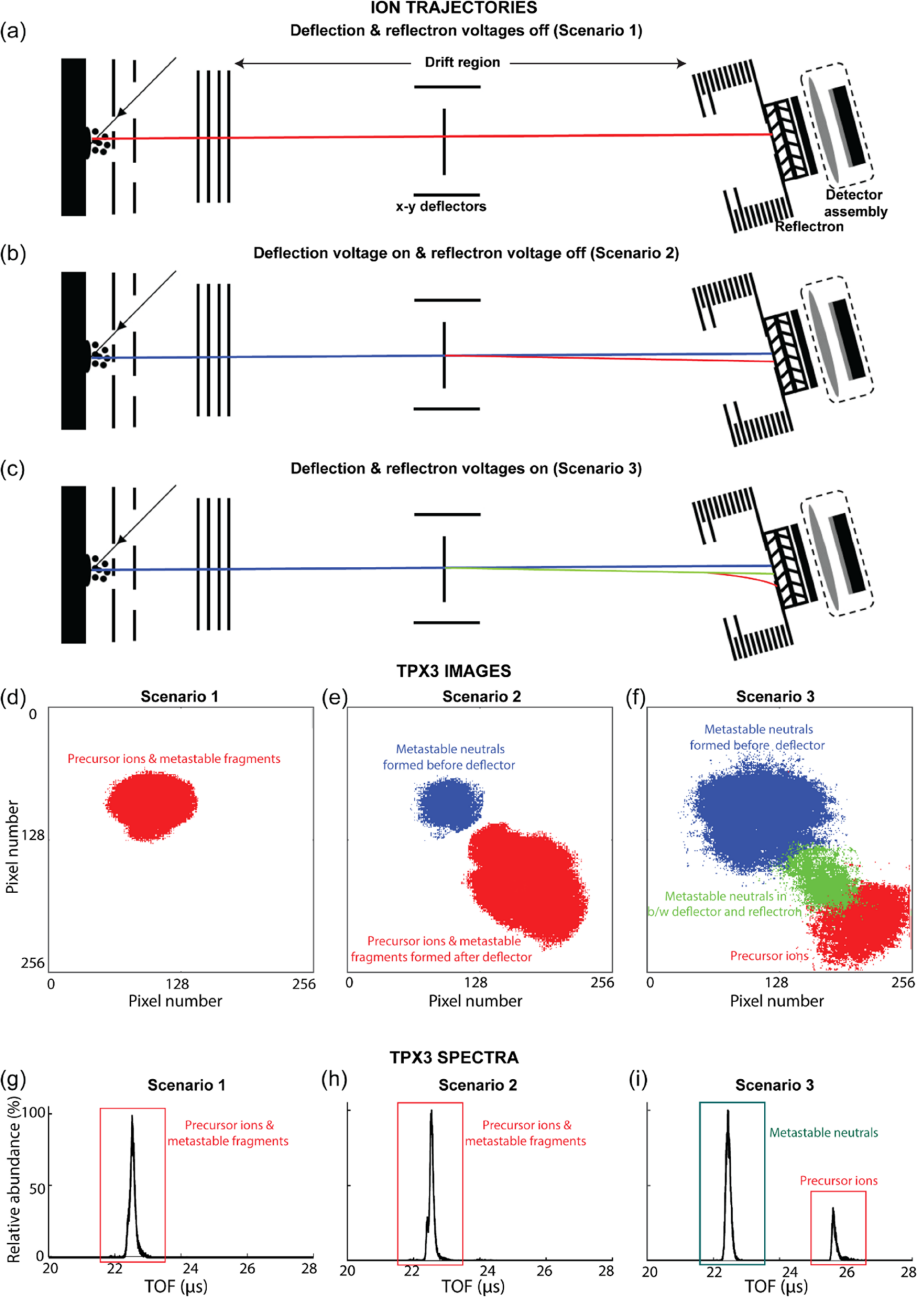
Ion trajectory (a–c) and the resulting total ion TPX3 image (d–f) and TOF spectrum (g–i) produced from insulin chain B [M + H]^1+^ ions (in CHCA matrix) for three different conditions; (1) without deflection and reflectron voltages, (2) with deflection voltage (*x* and *y* deflection voltages: −55 and −65 V), and (3) with deflection and reflectron voltages (*x* and *y* deflection voltages: −55 and −65 V, reflectron voltage: 19.5 kV). The ion trajectories and corresponding spatial distributions at the TPX3 detector of each type of ion cloud are represented using the same color in all three scenarios. The mass spectra were generated by the summation of the number of ion events over successive 1.5625 ns time windows in each TOF cycle for 5000 laser shots. The details on the plotting of TPX3 images and ion events spectrum are provided under the “Data Acquisition and Analysis” section. Data acquisition parameters are listed in Table S1 (Supporting Information).

All TPX3 spectra were recorded with a time resolution of 1.5625 ns. The “total pixels spectrum” (Figures 2c, 3b, and 4a,c,e) was generated by the summation of the number of activated pixels over successive 1.5625 ns time windows in each TOF cycle for a number of measurement cycles. The total pixels spectrum in Figure 3b was resampled to 100 ns. The “total TOT spectrum” (Figure 2d) was generated by the summation of the TOT values of the pixels triggered in each 1.5625 ns window in a TOF cycle over a number of measurement cycles. Figure 5g–i was generated by the summation of the number of ion events over successive 1.5625 ns time windows in each TOF cycle for a number of measurement cycles. For single ion counting, TOF cycles were divided into 500 ns (475 (dead time) + 25 (minimum TOT)) time slices. The time dimension was then removed to create a 2D binary subframe. MATLAB function “regionprops” was used to detect and measure the properties of connected areas (pixel clusters) in these subframes. The TOF of the centroid pixel in the detected pixel blob was used to add one at that TOF position in the reconstructed spectrum to build the “ion events spectrum.” The simulated TPX data (Figure 4) was produced from the TPX3 data by converting each frame (TOF cycle) to binary 256 × 256 data by considering the earliest TOA event and ignoring the rest for each pixel.

The “*m*/*z*-resolved TPX3 images” in Figure 2e were generated by summing up the number of pixels activated corresponding to each TOF/*m*/*z* value over a number of measurement cycles. The “pixel histograms” in Figure 4b,d,f were plotted by converting the 256 × 256 pixel array total ion image into a 1 × 65,536 row vector. To create Figure 5d–f, each of the ion clouds in the total ion image was extracted from the MATLAB figure using a binary mask and exported to GNU image manipulation program (GIMP), where each ion cloud was given a distinct color, and then all of the ion clouds were recombined.

The MCP and phosphor screen data (Figures 2a,b and 3a) collected at 100 MHz sampling rate on the oscilloscope (LeCroy LT372) were saved as text files using the Scope Explorer software (LeCroy Corporation, New York, USA). The MCP spectrum in Figure 3b was resampled to 100 ns.

## Results and Discussion

### Detection of High *m*/*z* Ions Using TPX3CAM

We initially acquired the MALDI-axial TOF mass spectra of IgG (∼150 kDa) to investigate the performance of the TPX3CAM assembly for the detection of high *m*/*z* ions. IgG is a covalently bounded heterotetramer comprised of two heavy chains (HC, ∼50 kDa) and two light chains (LC, ∼25 kDa), where the HCs are linked to each other and to a LC each by disulfide bonds.^35^Figure 2 shows the MALDI spectra of IgG acquired simultaneously from MCP (Figure 2a), phosphor screen (Figure 2b), and TPX3 (Figure 2c,d) channels by summing 5000 laser shots. Note that the analog signals registered on the MCP and phosphor screen are recorded at a 100 MHz digitation rate using an oscilloscope, whereas TPX3 data is acquired with a time resolution of 1.5625 ns. The singly charged IgG, doubly charged IgG, triply charged IgG, and singly charged LC ions were observed at *m*/*z* values of ∼150,000, ∼75,000, ∼50,000, and ∼25,000, respectively. The *m*/*z*-resolved TPX3 images of LC^1+^, IgG^3+^, IgG^2+^, and IgG^1+^ ions collected from 5000 laser shots are shown in Figure 2e. All *m*/*z* values are observed to have distorted elliptical spatial distributions with diameters that increase as the *m*/*z* value increases. This *m*/*z-*dependent focusing phenomena has previously been investigated in detail using MCP-TPX assembly on the same Ultraflex instrument.^11,14^

Both the MCP and phosphor screen spectra yield similar S/N and are comparable to the spectrum from the earlier studies using the conventional MCP/analog-to-digital converter (ADC) detection system on the Ultraflex.^11^ In Figure 2c, the TPX3 data has been plotted by summing the number of pixels activated across each 1.5625 ns TOA bin. Each ion impact on the detection assembly leads to a cascade of secondary electrons within the MCP that are in turn converted to photons by the scintillator and electron–hole pairs in the Si-coated TPX3 that result in a detectable current within an individual pixel. The footprint of a single ion event typically spans around an average of ∼4 pixels (Figure S2). This indicates that the detection of a single ion is effectively oversampled by a factor of 4 pixels in Figure 2c. This is consistent with earlier TPX detection of high *m*/*z* ions that yields significantly improved S/N values compared to the traditional MCP/ADC approach when using reduced MCP bias conditions.^11^ A new feature of the TPX3 is the capability to simultaneously record both TOA and TOT data for each pixel. The TOT corresponds to the time each pixel is over the threshold value required for event registration and is proportional to the number of electron–hole pairs generated. Figure 2d shows the TPX3 data represented in terms of the summed TOT values recorded for each TOA bin across 5000 laser shots. Typical TOT values for these experiments were ∼25–1050 (in ns) (Figure S3), which means each ion event is oversampled by a factor of 25–1050 (in ns) in Figure 2d. The significantly higher S/N ratio of TPX3 mass spectrum in Figure 2c,d compared to MCP and phosphorus screen spectrum is mainly due to the larger oversampling factor, which depends on various parameters such as MCP bias voltage, phosphor screen voltage, and camera aperture *f*-stop value.^20,21^

Next, we evaluated the performance of TPX3CAM at higher *m*/*z* values utilizing intact IgM (∼970 kDa). IgM exists as a pentamer, in which each of the five monomers is composed of two LCs (∼25 kDa) and two HCs (∼70 kDa). Monomers are bound together by disulfide bonds and a joining chain (∼15 kDa). Within the monomer, HCs are linked to each other and to a LC each by disulfide bonds.^36^Figure 3 shows the MALDI mass spectra of IgM simultaneously acquired using both the MCP (a) and TPX3 (b) channels for 5000 laser shots. The TPX3 spectrum is generated by summing up the number of pixels activated for each TOA bin (similar to the approach used for plotting Figure 2c). The MCP spectrum was recorded at a 100 MHz digitization rate using an oscilloscope, whereas TPX3 data was acquired using time bins of 1.5625 ns. Both spectra were resampled to 100 ns and baseline subtracted. Only the IgM 2^+^–5^+^ ions with *m*/*z* values of ∼485,000, ∼323,333, ∼242,500, and ∼194,000 were detected using the MCP signal with poor S/N ratios. In contrast, TPX3 data yields significantly higher S/N values and even detects singly charged IgM ions at an *m*/*z* of 970,000. The application of high MCP bias voltage (2 kV) and low TPX3CAM aperture *f*-stop value (0.95) generated large clusters with an average size of ∼18–37 pixels (Figure S4) and resulted in a high-quality TPX3CAM spectrum when compared to the MCP spectrum. Given the decrease in efficiency of the ion-to-electron conversion of MCPs with an increase in the *m*/*z* (decreasing velocity),^21,37−41^ the ability of the TPX3 to detect intact protein ions with *m*/*z* approaching 1,000,000 is remarkable and corresponds to ∼2.5 times increase in *m*/*z* range comparable to previous TPX studies, and its performance is comparable to other high mass detection systems such as CovalX HM detectors,^42^ cryogenic detectors based on superconducting tunnel junctions,^43−47^ calorimeters/bolometers, superconducting nanostripline detectors/nanomembrane detectors,^48^ and nanoelectromechanical system (NEMS) detectors.^49,50^

### Event-Based Readout Using TPX3CAM

The benefits of switching the readout from frame-based (TPX) to packet-based architecture (TPX3) is discussed in this section. Data from the earlier generation TPX chip is formatted in frames (TOF cycles) of binary data from all of the pixels, including pixels that did not activate during the measurement window. This results in a readout time >300 μs for each frame. In addition, the measurement had to be paused during the data readout of each frame in TPX. Due to these reasons, previous TPX studies had to be performed at reduced frame rates.^11,20^ Unlike the TPX chip, the on-chip zero suppression scheme implemented in the TPX3 chip allows only pixels with event data to be read out simultaneously during the data acquisition. This reduces the dead time per pixel (475 ns + TOT) and the total readout time of the pixel matrix for occupancies (% of pixel hits) below 50%.^1,2,51^ This unique data-driven readout system allows the TPX3 to operate at least 10 times faster compared to the previous Ultraflex-TPX experimental setup, where the data is read out by 1 Gbps in both cases.^11^ The 10 Gbps ethernet interface at the SPIDR readout system allows the instrument to operate with a higher speed, which would be beneficial in high-throughput mass spectrometry imaging experiments.^52,53^

Further, the single-stop pixels in the TPX limited the detection of higher *m*/*z* values, especially at high count rates (e.g., those encountered using MALDI MS) and high MCP voltages that lead to high pixel occupancies, as pixels are deactivated by lower *m*/*z* (earlier arriving) ions. In contrast, TPX3 is designed to enable multihit functionality such that each pixel can record multiple events within a single measurement cycle (in this case, for each laser shot). This makes TPX3 better suited for the analysis of complex mixtures and ensures that spectra better reflect the true abundances of ions generated. Figure 4 shows the mass spectra (a, c) and pixel histograms (b, d) plotted in event-based (TPX3, blue trace) and single-stop frame-based (simulated TPX, orange trace) modes for two different mass ranges: (a, b) Bruker peptide calibration standard II (*m*/*z* range: 700–3200 Da, case 1); and (c, d) IgM (*m*/*z* range: 190–970 kDa, case 2). The number of pixels activated per TOA bin is the sum of 1000 laser shots. The pixel parameters corresponding to the earliest TOA were used to simulate frame-based TPX data, and the rest were ignored in each TOF cycle. This has the effect of simulating the single-stop behavior of TPX. In both cases 1 and 2, the TPX3 data registers more events than the simulated TPX data due to its multihit capabilities. The green trace in Figure 4a,c shows the ratio of signal intensities in simulated TPX and TPX3 modes and shows a decreasing trend with an increase in *m*/*z* and thus demonstrates that utilizing TPX3 over TPX improves detection efficiency for higher *m*/*z* species when detected in the presence of ions with lower *m*/*z*. The average number of ion events per TOF cycle and pixel cluster size for case 1 are ∼2942 and ∼3–6 pixels (Figure S5), and for case 2, are ∼144 and ∼18–37 pixels (Figure S4). In case 1, the high count rate at low *m*/*z* causes some high *m*/*z* ions to be missed in TPX mode, as the pixels they strike may have been rendered inactive by the earlier arrival of lower *m*/*z* ions. Case 2 has a much lower number of ions arriving at the detector per TOF cycle than case 1; however, the pixel cluster size is larger due to the higher MCP bias voltage used for the detection of heavy ions, and thus each ion event occupies more pixels. The higher pixel occupancy by low *m*/*z* ions causes the inactivation of pixels when struck by high *m*/*z* ions and results in a low detection efficiency at high *m*/*z* in TPX mode. Figure 4e shows the mass spectrum produced from the Bruker protein calibration standard II (*m*/*z* range: 10–70 Da, case 3) plotted in event-based mode by the accumulation of 75 laser shots (blue trace) and in frame-based mode by the accumulation of 1000 TOF cycles (orange trace). Figure 4f shows the number of pixels activated for the same data shown in Figure 4e for 1000 laser shots, revealing again the additional events registered in TPX3 mode. Despite the reduced number of laser shots, the event-based TPX3 data in Figure 4e shows a similar signal intensity of the most intense peak (*m*/*z* = 23,983) as that of the simulated single-stop TPX data, again highlighting the benefits of the event-based TPX3 acquisition. This enhanced performance in TPX3 mode in case 3 is attributed to the fact that many different ions can strike the same pixels due to the large spatial overlap of the ion clouds of different *m*/*z* values (Figures 4f and S6).

### Visualization of Metastable Fragments Produced in the TOF Tube

The spatially resolved detection capability of TPX3CAM can be employed for the evaluation of fragments produced by metastable decay (post-source decay, PSD) at different locations within the field-free flight tube. Typically, in linear TOF mode, metastable fragments produced in the field-free region have flight times and impact positions identical to the precursor ion (ignoring small deviations caused by fragment recoil).^54−56^ Following MALDI, PSD will generally produce a singly charged ionic and neutral fragment pair (assuming a singly charged precursor). Here, we have combined the imaging capability of the linear TPX3 detector with an electrostatic deflector and retarding fields of the reflectron to spatially separate neutral fragments formed at different locations along the ion flight path in the field-free region.

The adjustable *x*–*y* deflectors positioned within the flight tube prior to the reflectron were used to spatially separate ions and neutral fragments formed between the source and deflector. The application of retarding field using the reflectron along with the deflector voltage leads to (i) temporal separation of ions and neutral fragments (ions shifted to longer TOF) and (ii) spatial separation of neutral fragments formed between the source and deflector and the deflector and reflectron. This spatial separation of the fragments arises as the neutral fragments produced after the deflectors travel on the same path as deflected ions until they reach the reflectron, whereas neutrals formed before the deflector travel on a path unperturbed by the deflection voltages. In addition, due to the greater residence time of ions in the reflectron, neutral fragments formed after the deflector are also spatially separated from intact ions. The combination of deflection and retarding voltages thus results in three impact zones, one corresponding to intact ions reaching the reflectron at longer TOF, and two impact regions corresponding to neutrals at different locations along the flight path (one group formed prior to the deflector, a second group formed in between the deflector and reflectron). This is exemplified in Figure 5.

Figure 5a,d,g shows the ion trajectory and the resulting TPX3 image and TOF spectrum produced from insulin chain B [M + H]^1+^ ions (in CHCA matrix) when no deflection or retarding fields are applied (scenario 1). Ion source voltages were adjusted to spatially focus the ion packet to a small circular cloud for all of the data acquisition in this section. However, it should be noted that these conditions are different from those needed for time focusing of the ions on the detector, and thus broader peaks are observed.^14^ As expected, these conditions yield one impact zone on the detector and one peak in the TOF spectrum corresponding to protonated insulin chain B. When the deflection voltage is activated (scenario 2, *x* and *y* deflection voltages: −55 and −65 V), the deflected ions strike closer toward the bottom right of the detector (Figure 5b,e, red), whereas neutral fragments formed prior to the deflector remain on their original trajectory (Figure 5b,e, blue). These conditions still result in a single TOF peak (Figure 5h). Smaller pixel clusters (a parameter that is related to the MCP detection efficiency) were observed for neutrals compared to the precursor ions. This is caused by a reduced ion-to-electron conversion efficiency when neutrals impact the MCP.^21^ The average pixel cluster size for metastable product neutrals and precursor ions are 3.5 and 5.5 pixels, respectively (Figure S7). Figure S8 (Supporting Information) compares the TOF spectra generated for precursor ions (red trace) and metastable neutrals (blue trace) from the TPX3 image. The result clearly shows that the metastable fragments arrive at a slightly later time compared to the precursor ions. This could be attributed to the postacceleration of ions at the end of the TOF tube because of the negative potential at the front MCP. When both the deflection and retarding fields are applied (scenario 3, *x* and *y* deflection voltages: −55 and −65 V, reflectron voltage: 19.5 kV), ions are further deflected due to the greater residence time in the reflectron (Figure 5c,f, red). Note that not the full reflectron voltage is applied but a reduced voltage, enough to deflect the ions slightly from their original flight path. An additional packet of neutrals (Figure 5c,f, green) is observed at the same location ions are observed to strike the detector in Figure 5b,e (red) that are assigned to neutral fragments formed by PSD events between the deflector and reflectron. The ions are shifted to longer TOA due to the retarding field, while neutrals are detected at the original ion TOA time (Figure 5i). Figure S9 (Supporting Information) shows the evolution of the total ion TPX3 images, linear TPX3 detector TOF spectra with an increase in the reflectron voltage (a deflection voltage is also applied). The square patterns that are only observed in the precursor ion clouds and not in the metastable product neutral clouds in the TPX3 images are explained by the transmission grids placed in the reflectron. The TOF of the ions increases with an increase in reflectron voltage as the ions spend more time in the reflectron, whereas the TOF of metastable neutrals is insensitive to the reflectron voltage. While outside the scope of this work, the unique ability of space and time-resolved detection to detect metastable fragments formed at different locations along the flight path provides an interesting avenue to study the kinetics of metastable ion decay.

## Conclusions

In this work, we have described the first implementation of a TPX3CAM detection assembly on a MALDI TOF (Ultraflex III) MS for the detection and ion imaging of high-mass biomolecules. This new experimental setup significantly extended the *m*/*z* range previously detected with the TPX family by the measurement of the intact protein ions of *m*/*z* approaching 1,000,000 Da. The enhanced time resolution, simultaneous measurement of TOT and TOA and multihit capabilities of the TPX3 chip compared to its predecessor, the TPX chip, allowed the generation of the TOF/mass spectra with a better S/N ratio and improved the sensitivity of the high *m*/*z* detection in the presence of low *m*/*z* ions at high count rates and detector voltages. The utilization of deflectors and time-resolved imaging capabilities of TPX3 allowed us to distinguish PSD events occurring at different locations along the flight path and provides a unique approach to explore the kinetics of PSD as well as the influence of both MALDI and parameters such as laser fluence, MALDI matrix, and extraction conditions on the metastable decay rate.^54,55,57−59^

## Supporting Information Available

List of the data acquisition parameters (Table S1); TOF to *m*/*z* conversion curve (Figure S1); pixel cluster histogram of IgG ions (Figure S2); TOT distribution of IgG ions (Figure S3); pixel cluster histogram of IgM ions (Figure S4); pixel cluster histogram of ions generated from Bruker peptide calibration standard II (Figure S5); *m*/*z-*resolved TPX3 images corresponding to the main peaks of Bruker protein calibration standard II mass spectrum (Figure S6); pixel cluster histograms of the precursor insulin chain B [M + H]^1+^ ions and metastable neutrals formed prior to the deflector (Figure S7); TOF spectra of the precursor insulin chain B [M + H]^1+^ ions and metastable neutrals formed prior to the deflector (Figure S8); and axial TPX3 images and TOF spectra of the precursor insulin chain B [M + H]^1+^ ions and metastable neutrals at different reflectron voltages (Figure S9) (PDF)

## References

[ref1] Llopart X.; Ballabriga R.; Campbell M.; Tlustos L.; Wong W. Timepix, a 65k programmable pixel readout chip for arrival time, energy and/or photon counting measurements. Nucl. Instrum. Methods Phys. Res., Sect. A 2007, 581, 485–494. 10.1016/j.nima.2007.08.079.

[ref2] Poikela T.; Plosila J.; Westerlund T.; Campbell M.; De Gaspari M.; Llopart X.; Gromov V.; Kluit R.; van Beuzekom M.; Zappon F.; Zivkovic V.; Brezina C.; Desch K.; Fu Y.; Kruth A. Timepix3: a 65K channel hybrid pixel readout chip with simultaneous ToA/ToT and sparse readout. J. Instrum. 2014, 9, C05013 10.1088/1748-0221/9/05/C05013.

[ref3] Llopart X.; Alozy J.; Ballabriga R.; Campbell M.; Casanova R.; Gromov V.; Heijne E.; Poikela T.; Santin E.; Sriskaran V.; et al. Timepix4, a large area pixel detector readout chip which can be tiled on 4 sides providing sub-200 ps timestamp binning. J. Instrum. 2022, 17, C01044 10.1088/1748-0221/17/01/C01044.

[ref4] Campbell M.; 10 years of the Medipix2 Collaboration. Nucl. Instrum. Methods Phys. Res., Sect. A 2011, 633, S1–S10. 10.1016/j.nima.2010.06.106. PMC3170860 21918588

[ref5] Ballabriga R.; Campbell M.; Llopart X. Asic developments for radiation imaging applications: The medipix and timepix family. Nucl. Instrum. Methods Phys. Res., Sect. A 2018, 878, 10–23. 10.1016/j.nima.2017.07.029.

[ref6] Tremsin A.; Vallerga J. Unique capabilities and applications of Microchannel Plate (MCP) detectors with Medipix/Timepix readout. Radiat. Meas. 2020, 130, 106228 10.1016/j.radmeas.2019.106228.

[ref7] Bamberger C.; Renz U.; Bamberger A. Digital imaging mass spectrometry. J. Am. Soc. Mass Spectrom. 2011, 22, 1079–1087. 10.1007/s13361-011-0120-1. 21953049

[ref8] Jungmann J. H.; MacAleese L.; Visser J.; Vrakking M. J.; Heeren R. M. High dynamic range bio-molecular ion microscopy with the Timepix detector. Anal. Chem. 2011, 83, 7888–7894. 10.1021/ac2017629. 21882854

[ref9] Jungmann J. H.; MacAleese L.; Buijs R.; Giskes F.; De Snaijer A.; Visser J.; Visschers J.; Vrakking M. J.; Heeren R. M. Fast, high resolution mass spectrometry imaging using a medipix pixelated detector. J. Am. Soc. Mass Spectrom. 2011, 21, 2023–2030. 10.1016/j.jasms.2010.08.014. 20971654

[ref10] Jungmann J. H.; Smith D. F.; MacAleese L.; Klinkert I.; Visser J.; Heeren R. M. Biological tissue imaging with a position and time sensitive pixelated detector. J. Am. Soc. Mass Spectrom. 2012, 23, 1679–1688. 10.1007/s13361-012-0444-5. 22836864

[ref11] Ellis S. R.; Jungmann J. H.; Smith D. F.; Soltwisch J.; Heeren R. M. Enhanced Detection of High-Mass Proteins by Using an Active Pixel Detector. Angew. Chem., Int. Ed. 2013, 52, 11261–11264. 10.1002/anie.201305501. 24039122

[ref12] Jungmann J. H.; Smith D. F.; Kiss A.; MacAleese L.; Buijs R.; Heeren R. M. A. An in-vacuum, pixelated detection system for mass spectrometric analysis and imaging of macromolecules. Int. J. Mass Spectrom. 2013, 341–342, 34–44. 10.1016/j.ijms.2013.02.010.

[ref13] Kiss A.; Smith D. F.; Jungmann J. H.; Heeren R. M. Cluster secondary ion mass spectrometry microscope mode mass spectrometry imaging. Rapid Commun. Mass Spectrom. 2013, 27, 2745–2750. 10.1002/rcm.6719. 24214859

[ref14] Ellis S. R.; Soltwisch J.; Heeren R. M. Time-resolved imaging of the MALDI linear-TOF ion cloud: direct visualization and exploitation of ion optical phenomena using a position- and time-sensitive detector. J. Am. Soc. Mass Spectrom. 2014, 25, 809–819. 10.1007/s13361-014-0839-6. 24658803

[ref15] Soltwisch J.; Goritz G.; Jungmann J. H.; Kiss A.; Smith D. F.; Ellis S. R.; Heeren R. M. MALDI mass spectrometry imaging in microscope mode with infrared lasers: bypassing the diffraction limits. Anal. Chem. 2014, 86, 321–325. 10.1021/ac403421v. 24308447

[ref16] Syed S. U. A. H.; Eijkel G. B.; Kistemaker P.; Ellis S.; Maher S.; Smith D. F.; Heeren R. M. Experimental investigation of the 2D ion beam profile generated by an ESI octopole-QMS system. J. Am. Soc. Mass Spectrom. 2014, 25, 1780–1787. 10.1007/s13361-014-0958-0. 25113629

[ref17] Syed S. U. A. H.; Eijkel G. B.; Maher S.; Kistemaker P.; Taylor S.; Heeren R. M. A micropixelated ion-imaging detector for mass resolution enhancement of a QMS instrument. Anal. Bioanal. Chem. 2015, 407, 2055–2062. 10.1007/s00216-014-8158-0. 25270865

[ref18] Syed S. U. A. H.; Maher S.; Eijkel G. B.; Ellis S. R.; Jjunju F.; Taylor S.; Heeren R. M. Direct ion imaging approach for investigation of ion dynamics in multipole ion guides. Anal. Chem. 2015, 87, 3714–3720. 10.1021/ac5041764. 25710191

[ref19] Jenčič B.; Sepec L.; Vavpetic P.; Kelemen M.; Rupnik Z.; Vencelj M.; Vogel-Mikus K.; Potocnik N. O.; Ellis S. R.; Heeren R.; Pelicon P. Stigmatic imaging of secondary ions in MeV-SIMS spectrometry by linear Time-of-Flight mass spectrometer and the TimePix detector. Nucl. Instrum. Methods Phys. Res., Sect. A 2019, 452, 1–6. 10.1016/j.nimb.2019.05.040.

[ref20] Mathew A.; Buijs R.; Eijkel G. B.; Giskes F.; Dyachenko A.; van der Horst J.; Byelov D.; Spaanderman D. J.; Heck A. J. R.; Porta Siegel T.; Ellis S. R.; Heeren R. M. A. Ion Imaging of Native Protein Complexes Using Orthogonal Time-of-Flight Mass Spectrometry and a Timepix Detector. J. Am. Soc. Mass Spectrom. 2021, 32, 569–580. 10.1021/jasms.0c00412. 33439014 PMC7863068

[ref21] Mathew A.; Eijkel G. B.; Anthony I. G.; Ellis S. R.; Heeren R. M. Characterization of Microchannel Plate Detector Response for the Detection of Native Multiply Charged High Mass Single Ions in Orthogonal-Time-of-Flight Mass Spectrometry Using a Timepix Detector. J. Mass Spectrom. 2022, 57, e4820 10.1002/jms.4820. 35347816 PMC9287041

[ref22] Fisher-Levine M.; Nomerotski A. TimepixCam: a fast optical imager with time-stamping. J. Instrum. 2016, 11, C03016 10.1088/1748-0221/11/03/C03016.

[ref23] Nomerotski A.; Chakaberia I.; Fisher-Levine M.; Janoska Z.; Takacs P.; Tsang T. Characterization of TimepixCam, a fast imager for the time-stamping of optical photons. J. Instrum. 2017, 12, C01017 10.1088/1748-0221/12/01/C01017.

[ref24] Nomerotski A. Imaging and time stamping of photons with nanosecond resolution in Timepix based optical cameras. Nucl. Instrum. Methods Phys. Res., Sect. A 2019, 937, 26–30. 10.1016/j.nima.2019.05.034.

[ref25] Zhao A.; van Beuzekom M.; Bouwens B.; Byelov D.; Chakaberia I.; Cheng C.; Maddox E.; Nomerotski A.; Svihra P.; Visser J.; Vrba V.; Weinacht T. Coincidence velocity map imaging using Tpx3Cam, a time stamping optical camera with 1.5 ns timing resolution. Rev. Sci. Instrum. 2017, 88, 113104 10.1063/1.4996888. 29195350

[ref26] Fisher-Levine M.; Boll R.; Ziaee F.; Bomme C.; Erk B.; Rompotis D.; Marchenko T.; Nomerotski A.; Rolles D. Time-resolved ion imaging at free-electron lasers using TimepixCam. J. Synchrotron Radiat. 2018, 25, 336–345. 10.1107/S1600577517018306. 29488911

[ref27] Allum F.; Cheng C.; Howard A. J.; Bucksbaum P. H.; Brouard M.; Weinacht T.; Forbes R. Multi-Particle Three-Dimensional Covariance Imaging:“Coincidence” Insights into the Many-Body Fragmentation of Strong-Field Ionized D2O. J. Phys. Chem. Lett. 2021, 12, 8302–8308. 10.1021/acs.jpclett.1c02481. 34428066

[ref28] Lam H. V. S.; Yarlagadda S.; Venkatachalam A.; Wangjam T. N.; Kushawaha R. K.; Cheng C.; Svihra P.; Nomerotski A.; Weinacht T.; Rolles D.; Kumarappan V. Angle-dependent strong-field ionization and fragmentation of carbon dioxide measured using rotational wave packets. Phys. Rev. A 2020, 102, 043119 10.1103/PhysRevA.102.043119.

[ref29] Cheng C.; Forbes R.; Howard A. J.; Spanner M.; Bucksbaum P. H.; Weinacht T. Momentum-resolved above-threshold ionization of deuterated water. Phys. Rev. A 2020, 102, 052813 10.1103/PhysRevA.102.052813.

[ref30] Liu Y.; Rozgonyi T.; Marquetand P.; Weinacht T. Excited-state dynamics of CH2I2 and CH2IBr studied with UV-pump VUV-probe momentum-resolved photoion spectroscopy. J. Chem. Phys. 2020, 153, 184304 10.1063/5.0026177. 33187419

[ref31] Cheng C.; Vindel-Zandbergen P.; Matsika S.; Weinacht T. Electron correlation in channel-resolved strong-field molecular double ionization. Phys. Rev. A 2019, 100, 053405 10.1103/PhysRevA.100.053405.

[ref32] Debrah D. A.; Stewart G. A.; Basnayake G.; Nomerotski A.; Svihra P.; Lee S. K.; Li W. Developing a camera-based 3D momentum imaging system capable of 1 Mhits/s. Rev. Sci. Instrum. 2020, 91, 023316 10.1063/1.5138731. 32113393

[ref33] Visser J.; Van Beuzekom M.; Boterenbrood H.; Van Der Heijden B.; Muñoz J.; Kulis S.; Munneke B.; Schreuder F. SPIDR: a read-out system for Medipix3 & Timepix3. J. Instrum. 2015, 10, C12028 10.1088/1748-0221/10/12/C12028.

[ref34] Winter B.; King S.; Brouard M.; Vallance C. A fast microchannel plate-scintillator detector for velocity map imaging and imaging mass spectrometry. Rev. Sci. Instrum. 2014, 85, 023306 10.1063/1.4866647. 24593353

[ref35] Vidarsson G.; Dekkers G.; Rispens T. IgG subclasses and allotypes: from structure to effector functions. Front. Immunol. 2014, 5, 520 10.3389/fimmu.2014.00520. 25368619 PMC4202688

[ref36] Li Y.; Wang G.; Li N.; Wang Y.; Zhu Q.; Chu H.; Wu W.; Tan Y.; Yu F.; Su X.-D.; et al. Structural insights into immunoglobulin M. Science 2020, 367, 1014–1017. 10.1126/science.aaz5425. 32029689

[ref37] Geno P.; Macfarlane R. Secondary electron emission induced by impact of low-velocity molecular ions on a microchannel plate. Int. J. Mass Spectrom. Ion Processes 1989, 92, 195–210. 10.1016/0168-1176(89)83028-9.

[ref38] Meier R.; Eberhardt P. Velocity and ion species dependence of the gain of microchannel plates. Int. J. Mass Spectrom. Ion Processes 1993, 123, 19–27. 10.1016/0168-1176(93)87050-3.

[ref39] Westmacott G.; Frank M.; Labov S.; Benner W. Using a superconducting tunnel junction detector to measure the secondary electron emission efficiency for a microchannel plate detector bombarded by large molecular ions. Rapid Commun. Mass Spectrom. 2000, 14, 1854–1861. 10.1002/1097-0231(20001015)14:19<1854::AID-RCM102>3.0.CO;2-M. 11006596

[ref40] Liu R.; Li Q.; Smith L. M. Detection of large ions in time-of-flight mass spectrometry: effects of ion mass and acceleration voltage on microchannel plate detector response. J. Am. Soc. Mass Spectrom. 2014, 25, 1374–1383. 10.1007/s13361-014-0903-2. 24789774 PMC4108536

[ref41] Chen X.; Westphall M. S.; Smith L. M. Mass spectrometric analysis of DNA mixtures: instrumental effects responsible for decreased sensitivity with increasing mass. Anal. Chem. 2003, 75, 5944–5952. 10.1021/ac030127h. 14588036

[ref42] Wenzel R.; Rohling U.; Nazabal A.; Hillenkamp F. Detector Device for High Mass Ion Detection, A Method for Analyzing Ions of High Mass and A Device for Selection between Ion Detectors. Google Patents, 2013.

[ref43] Wenzel R. J.; Matter U.; Schultheis L.; Zenobi R. Analysis of megadalton ions using cryodetection MALDI time-of-flight mass spectrometry. Anal. Chem. 2005, 77, 4329–4337. 10.1021/ac0482054. 16013843

[ref44] Ohkubo M.; Shigeri Y.; Kinumi T.; Saito N.; Ukibe M.; Chen Y.; Kushino A.; Kurokawa A.; Sato H.; Ichimura S. Fragmentation analysis by superconducting ion detectors in matrix-assisted laser desorption/ionization (MALDI). Nucl. Instrum. Methods Phys. Res., Sect. A 2006, 559, 779–781. 10.1016/j.nima.2005.12.137.

[ref45] Aksenov A. A.; Bier M. E. The analysis of polystyrene and polystyrene aggregates into the mega dalton mass range by cryodetection MALDI TOF MS. J. Am. Soc. Mass Spectrom. 2008, 19, 219–230. 10.1016/j.jasms.2007.10.019. 18083529

[ref46] Plath L. D.; Ozdemir A.; Aksenov A. A.; Bier M. E. Determination of iron content and dispersity of intact ferritin by superconducting tunnel junction cryodetection mass spectrometry. Anal. Chem. 2015, 87, 8985–8993. 10.1021/acs.analchem.5b02180. 26266697

[ref47] Sipe D. M.; Plath L. D.; Aksenov A. A.; Feldman J. S.; Bier M. E. Characterization of mega-dalton-sized nanoparticles by superconducting tunnel junction cryodetection mass spectrometry. ACS Nano 2018, 12, 2591–2602. 10.1021/acsnano.7b08541. 29481053

[ref48] Park J.; Aksamija Z.; Shin H.-C.; Kim H.; Blick R. H. Phonon-assisted field emission in silicon nanomembranes for time-of-flight mass spectrometry of proteins. Nano Lett. 2013, 13, 2698–2703. 10.1021/nl400873m. 23621694

[ref49] Sage E.; Brenac A.; Alava T.; Morel R.; Dupré C.; Hanay M. S.; Roukes M. L.; Duraffourg L.; Masselon C.; Hentz S. Neutral particle mass spectrometry with nanomechanical systems. Nat. Commun. 2015, 6, 6482 10.1038/ncomms7482. 25753929 PMC4366497

[ref50] Dominguez-Medina S.; Fostner S.; Defoort M.; Sansa M.; Stark A.-K.; Halim M. A.; Vernhes E.; Gely M.; Jourdan G.; Alava T.; et al. Neutral mass spectrometry of virus capsids above 100 megadaltons with nanomechanical resonators. Science 2018, 362, 918–922. 10.1126/science.aat6457. 30467165

[ref51] Wong W.; Alozy J.; Ballabriga R.; Campbell M.; Kremastiotis I.; Llopart X.; Poikela T.; Sriskaran V.; Tlustos L.; Turecek D. Introducing Timepix2, a frame-based pixel detector readout ASIC measuring energy deposition and arrival time. Radiat. Meas. 2019, 106230 10.1016/j.radmeas.2019.106230.

[ref52] Körber A.; Keelor J. D.; Claes B. S.; Heeren R. M.; Anthony I. G. Fast Mass Microscopy: Mass Spectrometry Imaging of a Gigapixel Image in 34 Minutes. Anal. Chem. 2022, 94, 14652 10.1021/acs.analchem.2c02870. 36223179 PMC9607864

[ref53] Wood D.; Burleigh R. J.; Smith N.; Bortoletto D.; Brouard M.; Burt M.; Nomerotski A.; Plackett R.; Shipsey I. Ion Microscope Imaging Mass Spectrometry Using a Timepix3-Based Optical Camera. J. Am. Soc. Mass Spectrom. 2022, 33, 2328 10.1021/jasms.2c00223. 36383767 PMC9732873

[ref54] Karas M.; Bahr U.; Strupat K.; Hillenkamp F.; Tsarbopoulos A.; Pramanik B. N. Matrix dependence of metastable fragmentation of glycoproteins in MALDI TOF mass spectrometry. Anal. Chem. 1995, 67, 675–679. 10.1021/ac00099a029.

[ref55] Spengler B. Post-source decay analysis in matrix-assisted laser desorption/ionization mass spectrometry of biomolecules. J. Mass Spectrom. 1997, 32, 1019–1036. 10.1002/(SICI)1096-9888(199711)32:10<1019::AID-JMS595>3.0.CO;2-G.

[ref56] Laskin J.; Lifshitz C. Kinetic energy release distributions in mass spectrometry. J. Mass Spectrom. 2001, 36, 459–478. 10.1002/jms.164. 11391803

[ref57] Brown R. S.; Carr B. L.; Lennon J. J. Factors that influence the observed fast fragmentation of peptides in matrix-assisted laser desorption. J. Am. Soc. Mass Spectrom. 1996, 7, 225–232. 10.1016/1044-0305(95)00676-1. 24203293

[ref58] Szilágyi Z.; Varney J. E.; Derrick P. J.; Vékey K. Dependence of matrix-assisted laser desorption/ionization post-source decay spectra on laser power. Rapid Commun. Mass Spectrom. 1998, 12, 489–492. 10.1002/(SICI)1097-0231(19980430)12:8<489::AID-RCM187>3.0.CO;2-H.

[ref59] Gabelica V.; Schulz E.; Karas M. Internal energy build-up in matrix-assisted laser desorption/ionization. J. Mass Spectrom. 2004, 39, 579–593. 10.1002/jms.651. 15236295

