# Supplementary material for: Time-Resolved Imaging of High Mass Proteins and Metastable Fragments Using Matrix-Assisted Laser Desorption/Ionization, Axial Time-of-Flight Mass Spectrometry, and TPX3CAM
Source: Anal Chem. 2022 Dec 27;95(2):1470–9. doi: 10.1021/acs.analchem.2c04480 (PMC9850352; doi:10.1021/acs.analchem.2c04480)
Supplement: Supplementary file 1 — ac2c04480_si_001.pdf [file ac2c04480_si_001.pdf]

# Supporting Information

# Time-Resolved Imaging of High Mass Proteins and Metastable Fragments Using Matrix-Assisted Laser Desorption/Ionization, Axial Time-of-Flight Mass Spectrometry, and TPX3CAM

Anjusha Mathew[1], Joel D. Keelor[2], Gert B. Eijkel[1], Ian G. M. Anthony[1], Jingming Long[2], Jord Prangsma[2], Ron M. A. Heeren[1*] and Shane R. Ellis[1,3*]

[1]Maastricht MultiModal Molecular Imaging (M4i) Institute, Division of Imaging Mass Spectrometry (IMS), Maastricht University, 6229 ER Maastricht, The Netherlands

[2]Amsterdam Scientific Instruments (ASI), Science Park 106, 1098 XG Amsterdam, The Netherlands

[3]Molecular Horizons and School of Chemistry and Molecular Bioscience, University of Wollongong, NSW 2522, Wollongong, Australia

*To whom correspondence should be addressed:

r.heeren@maastrichtuniversity.nl

sellis@uow.edu.au

## Table of contents

**Table S1** List of the data acquisition parameters........S-3
**Figure S1** TOF to *m/z* conversion curve........S-4
**Figure S2** Pixel cluster histogram of IgG ions........S-5
**Figure S3** TOT distribution of IgG ions........S-6
**Figure S4** Pixel cluster histogram of IgM ions........S-7
**Figure S5** Pixel cluster histogram of ions generated from Bruker peptide calibration standard II........S-8
**Figure S6** *m/z* resolved TPX3 images corresponding to the main peaks of Bruker protein calibration standard II mass spectrum........S-9
**Figure S7** Pixel cluster histograms of the precursor insulin chain B $[M+H]^{1+}$ ions and metastable neutrals formed prior to the deflector........S-10
**Figure S8** TOF spectra of the precursor insulin chain B $[M+H]^{1+}$ions and metastable neutrals formed prior to the deflector........S-11
**Figure S9** Axial TPX3 images and TOF spectra of the precursor insulin chain B $[M+H]^{1+}$ions and metastable neutrals at different reflectron voltages........S-12

**Table S1**. List of the data acquisition parameters

| Parameter | Figure 2 (IgG) | Figure 3 (IgM) | Figure 4a & b (Peptide std II) | Figure 4e & f (Protein std II) | Figure 5 (Insulin chain B) | Figure S9 (Insulin chain B) |
|---|---|---|---|---|---|---|
| Number of laser shots/measurement cycles | 5000 | 5000 | 1000 | 1000 | 5000 | 5000 |
| Laser repetition rate (Hz) | 100 | 100 | 100 | 100 | 10 | 10 |
| Laser power (%) | 50 | 70 | 30 | 50 | 30 | 30 |
| Target plate voltage (kV) | 25 | 25 | 25 | 25 | 25 | 25 |
| Second plate voltage (kV) | 21 | 21 | 23.6 | 23.1 | 22.45 | 22.45 |
| Lens voltage (kV) | 12 | 12 | 7 | 6 | 6 | 6 |
| Reflectron voltage (kV) | 0 | 0 | 0 | 0 | 0, 0 and 19.5 (Scenario 1, 2 and 3) | 0, 5, 10, 15, 20, 25 |
| Pulsed ion extraction (PIE, ns) | 500 | 2000 | 0 | 500 | 0 | 0 |
| Matrix suppression deflection (Da) | 15000 | 100000 | 700 | 10000 | 1000 | 1000 |
| Global attenuator offset (%) | 65 | 65 | 45 | 65 | 45 | 45 |
| Attenuator offset (%) | 15 | 15 | 25 | 15 | 25 | 25 |
| Focus offset (%) | 0 | 0 | 0 | 0 | 0 | 0 |
| Focus range (%) | 100 | 100 | 100 | 100 | 100 | 100 |
| Focus position (%) | 33 | 33 | 33 | 33 | 33 | 33 |
| x-deflection voltage (V) | 0 | 0 | 0 | 0 | 0, -55 and -55 (Scenario 1, 2 and 3) | -55 |
| y-deflection voltage (V) | 0 | 0 | 0 | 0 | 0, -65 and -65 (Scenario 1, 2 and 3) | -70 |
| Linear MCP front plate voltage (V) | -2150 | -2600 | -2100 | -2200 | -2100 | -2100 |
| Linear MCP back plate voltage (V) | -600 | -600 | -600 | -600 | -600 | -600 |
| Phosphor screen voltage (V) | 5000 | 5000 | 5000 | 5000 | 5000 | 5000 |
| TPX3CAM f-stop value | 0.95 | 0.95 | 0.95 | 0.95 | 0.95 | 0.95 |
| DG535 channel A pulse width (delay for TPX3, µs) | 75 | 125 | 20 | 20 | 20 | 20 |
| DG535 channel B pulse width (measurement window for TPX3, µs) | 1000 | 2000 | 150 | 500 | 100 | 100 |
| DG535 channel C pulse width (delay for internal TDC SPIDR, µs) | 75 | 125 | 20 | 20 | 20 | 20 |
| DG535 channel D pulse width (measurement window of internal TDC SPIDR, µs) | 100 | 100 | 100 | 100 | 100 | 100 |

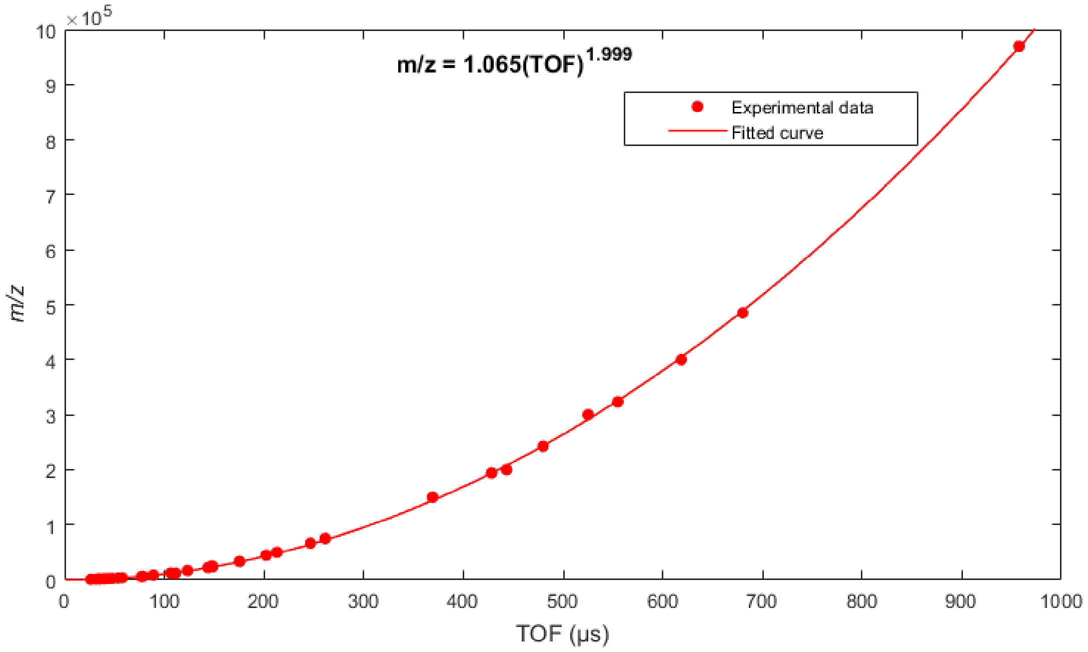


**Figure S1**. TOF to *m/z* conversion curve plotted using 10 samples that encompasses an *m/z* range from 750 to 970,000 Da. All the TOF data were acquired using an initial acceleration voltage (target plate voltage) of 25 kV.

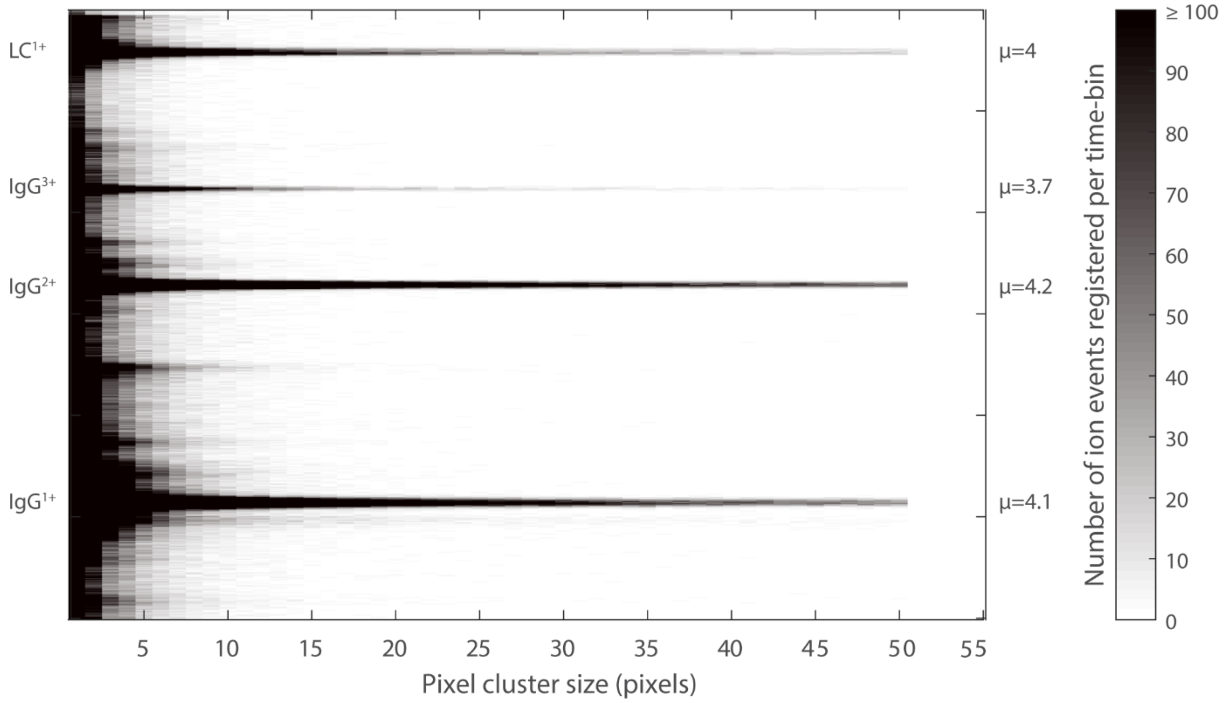


**Figure S2**. Distribution of pixel cluster area (in pixels) of $LC^{1+}$, $IgG^{3+}$, $IgG^{2+}$ and $IgG^{1+}$ ions, and corresponds to the data shown in Figure 2. A time-bin size of 500 ns was used for the generation of the histogram. μ is the average pixel cluster size that is calculated by $\frac{\sum_{i=1}^{k}(i\ x\ N_i)}{\sum_{i=1}^{k} N_i}$, where i=pixel cluster size (in pixels), $N_i$=number of ion events with pixel cluster size of 'i', and k= maximum pixel cluster size (in pixels).

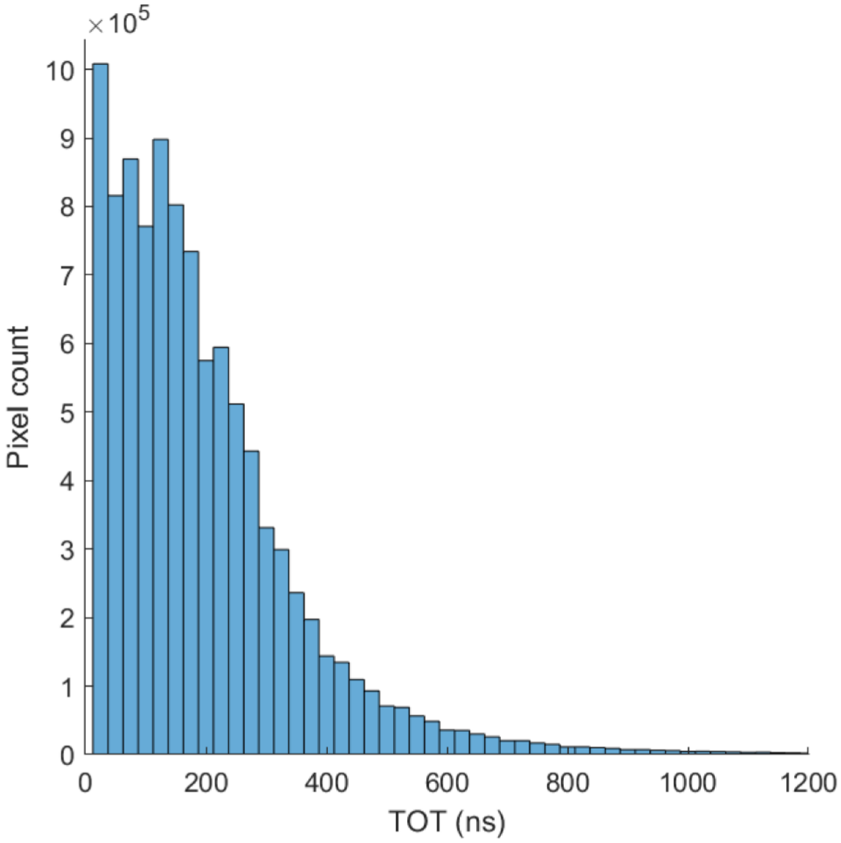


**Figure S3**. TOT distribution of the TPX3 pixels triggered by IgG ions (*m/z* range: 25-150 kDa), and corresponds to the data shown in Figure 2. A time-bin size of 25 ns was used for the generation of the histogram.

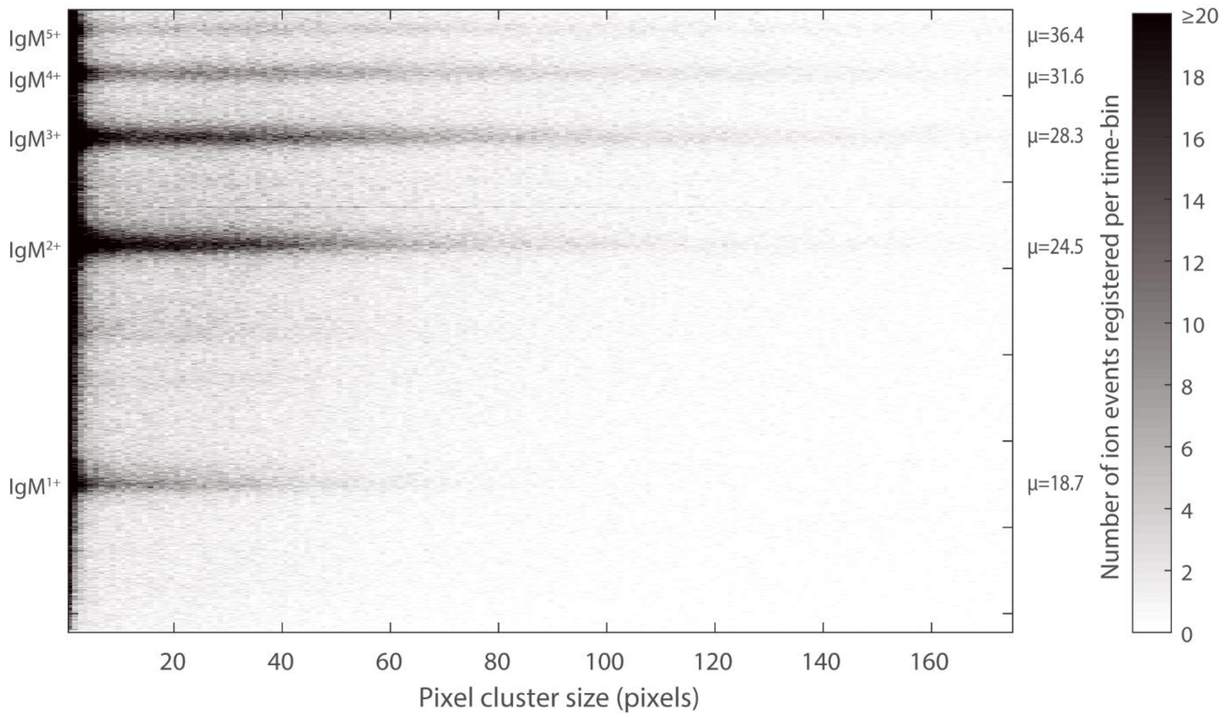


**Figure S4**. Distribution of pixel cluster area (in pixels) of IgM $1^{+}$-$5^{+}$ ions, and corresponds to the data shown in Figure 3. A time-bin size of 500 ns was used for the generation of the histogram. μ is the average pixel cluster size that is calculated as previously described (Figure S2).

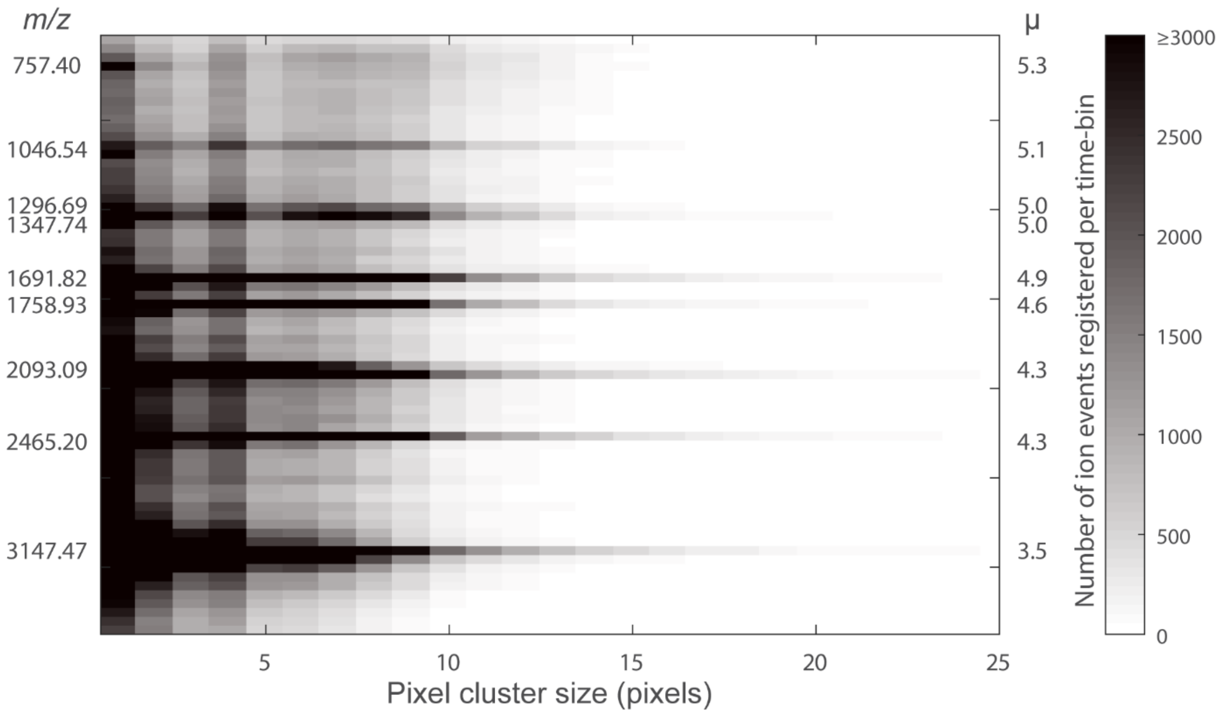


**Figure S5**. Distribution of pixel cluster area (in pixels) of the ions generated from Bruker peptide calibration standard II (*m/z* range: 700-3200 Da), and corresponds to data shown in Figure 4a and b. A time-bin size of 500 ns was used for the generation of the histogram. μ is the average pixel cluster size that is calculated as previously described (Figure S2).

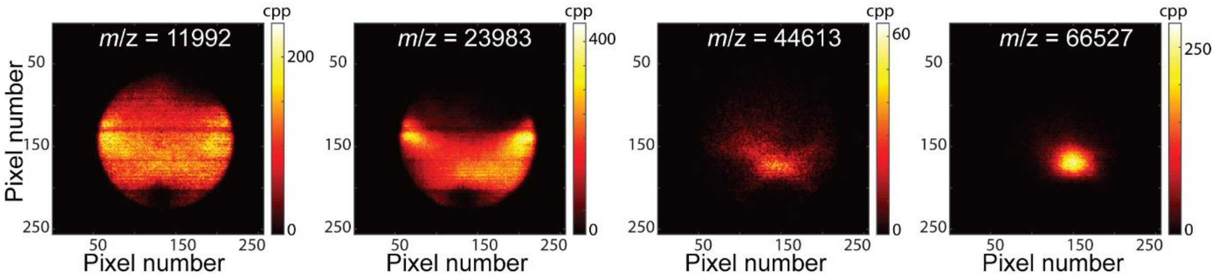


**Figure S6**. *m/z* resolved TPX3 images of Trypsinogen$^{2+}$ (*m/z*=11,992), Trypsinogen$^{1+}$ (*m/z*=23,983), Protein A$^{1+}$ (*m/z*=44,613) and BSA$^{1+}$ (*m/z*=66,527) from Bruker protein calibration standard II, and generated from data shown in Figure 4e and f by the accumulation of 1000 laser shots (cpp=counts per pixel).

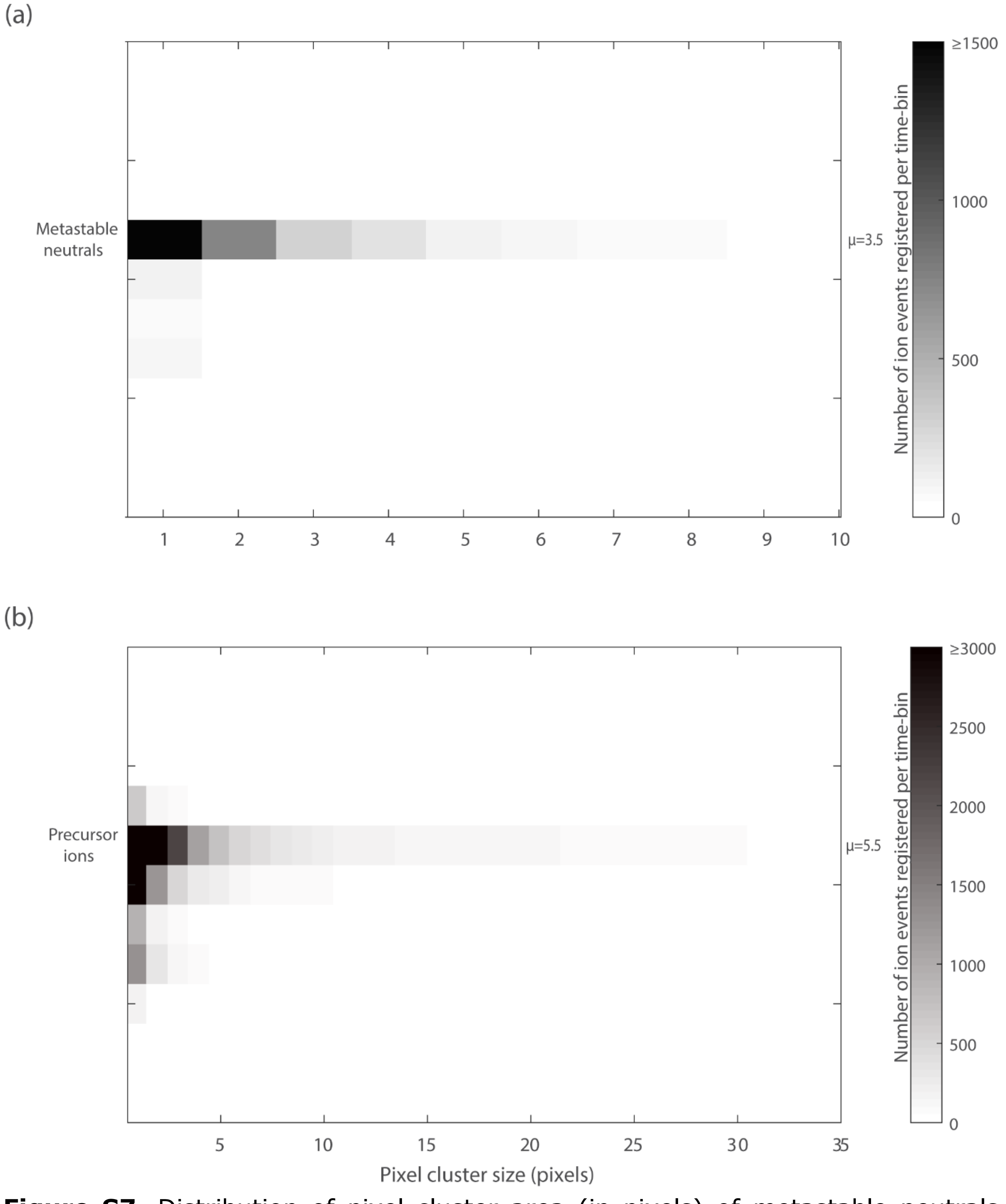


**Figure S7**. Distribution of pixel cluster area (in pixels) of metastable neutrals formed in between the source and deflector (a) and precursor insulin chain B $[M+H]^{1+}$ ions (b), and corresponds to data shown in Figure 5b, e and h (Scenario 2). A time-bin size of 500 ns was used for the generation of the histogram. μ is the average pixel cluster size that is calculated as previously described (Figure S2).

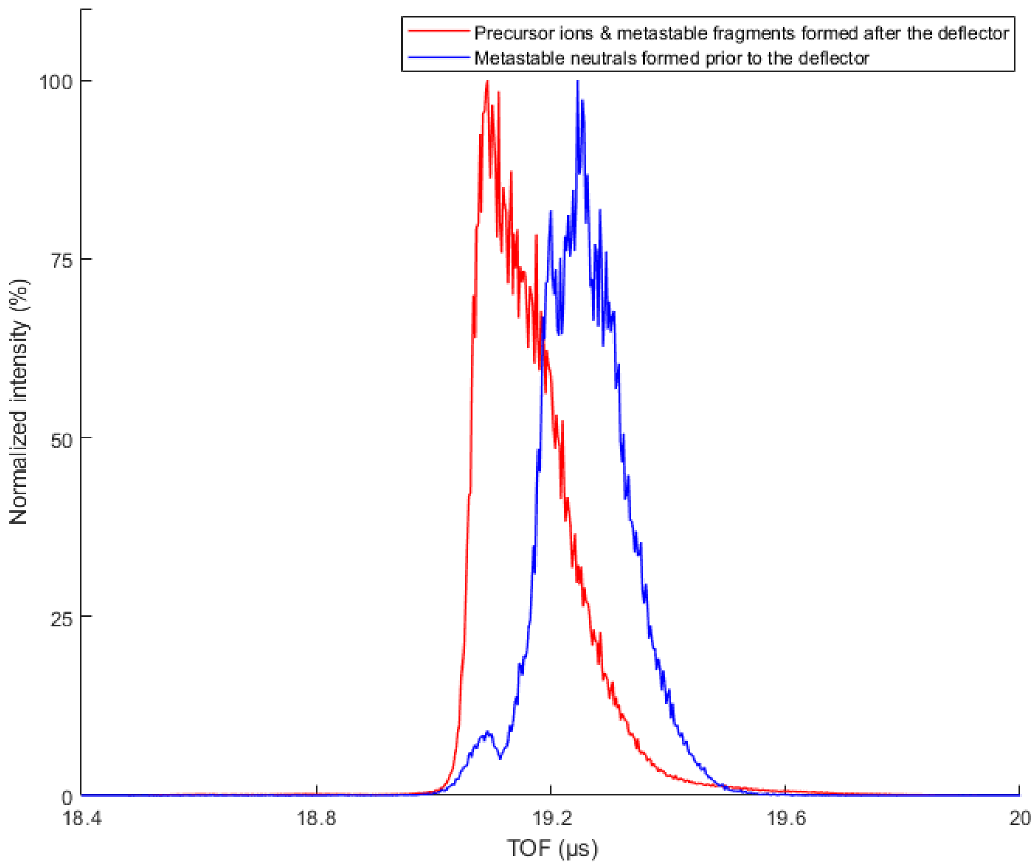


**Figure S8**. TOF spectra correspond to the intact insulin chain B $[M+H]^{1+}$ions (red trace) and metastable neutrals formed prior to the deflector (blue trace). Data was acquired with deflector voltage on and reflectron voltage off (Scenario 2 in Figure 5). Note that the intensities in both cases are normalized to their respective maximum for the better comparison and visualization of the TOF spectra.

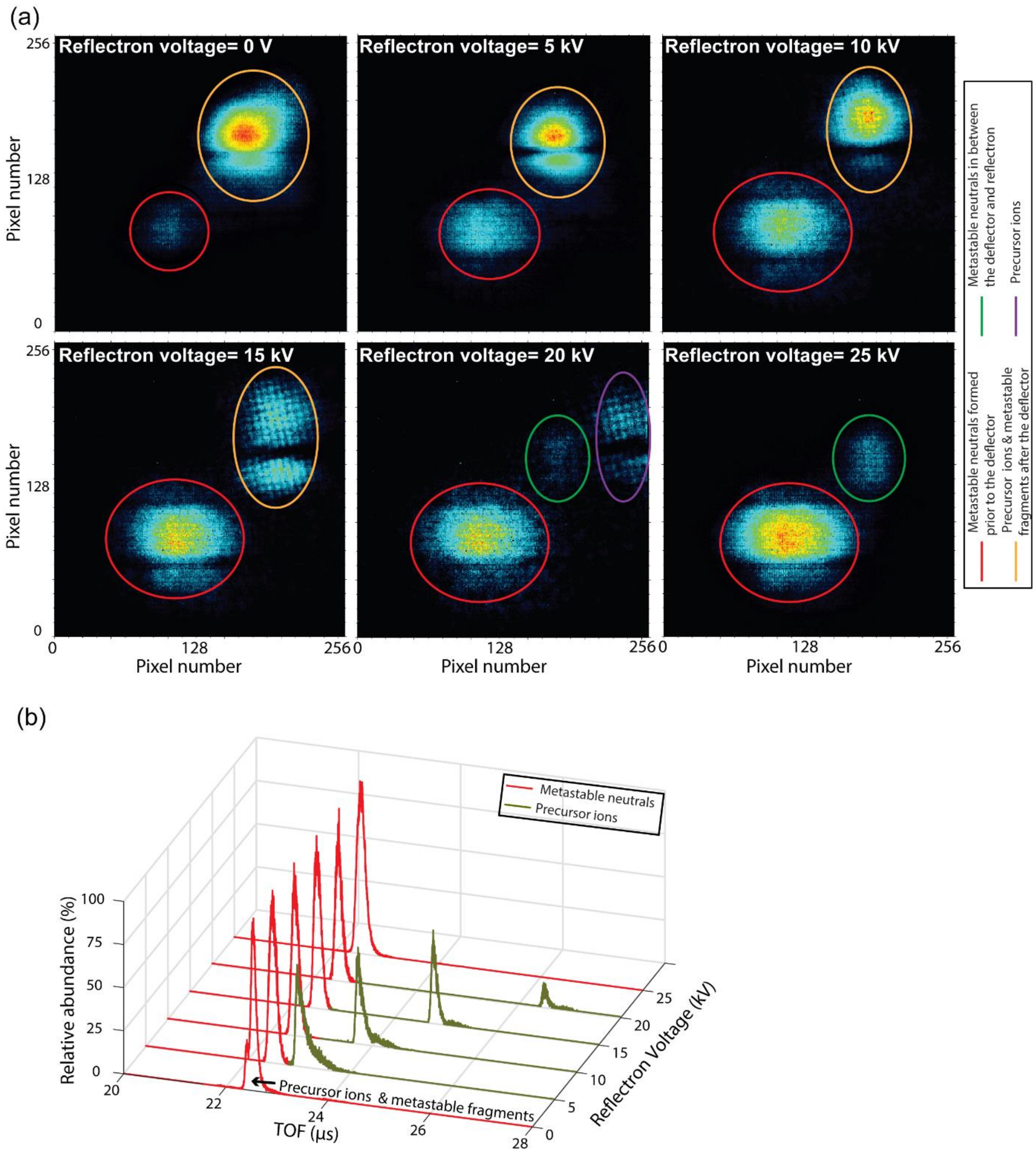


**Figure S9**. Evolution of the (a) total ion TPX3 images and (b) linear TPX3 detector TOF spectra with an increase in the reflectron voltage (a deflection voltage is also applied). Note that the intensities are normalized to their respective maximum for all the TOF spectra. The data acquisition parameters are listed in Table S1.
